# Sex-Specific Association of the X Chromosome With Cognitive Change and Tau Pathology in Aging and Alzheimer Disease

**DOI:** 10.1001/jamaneurol.2021.2806

**Published:** 2021-08-23

**Authors:** Emily J. Davis, Caroline W. Solsberg, Charles C. White, Elena Miñones-Moyano, Marina Sirota, Lori Chibnik, David A. Bennett, Philip L. De Jager, Jennifer S. Yokoyama, Dena B. Dubal

**Affiliations:** 1 Department of Neurology, Weill Institute for Neurosciences, University of California, San Francisco, San Francisco; 2 Biomedical Sciences Graduate Program, University of California, San Francisco, San Francisco; 3 Pharmaceutical Sciences and Pharmacogenetics Graduate Program, University of California, San Francisco, San Francisco; 4 Bakar Computational Health Sciences Institute, University of California, San Francisco, San Francisco; 5 Memory and Aging Center, University of California, San Francisco, San Francisco; 6 Rush Alzheimer’s Disease Center, Rush University Medical Center, Chicago, Illinois; 7 Center for Translational and Computational Neuroimmunology, Department of Neurology, Columbia University Medical Center, New York, New York; 8 Department of Pediatrics, University of California, San Francisco, San Francisco; 9 Biostatistics Center, Department of Neurology, Massachusetts General Hospital and Harvard Medical School, Boston; 10 Department of Epidemiology, Harvard T. H. Chan School of Public Health, Boston, Massachusetts; 11 Department of Radiology and Biomedical Imaging, University of California, San Francisco, San Francisco; 12 Associate Editor, *JAMA Neurology*

## Abstract

**Importance:**

The X chromosome represents 5% of the human genome in women and men, and its influence on cognitive aging and Alzheimer disease (AD) is largely unknown.

**Objective:**

To determine whether the X chromosome is associated with sex-specific cognitive change and tau pathology in aging and AD.

**Design, Setting, Participants:**

This study examined differential gene expression profiling of the X chromosome from an RNA sequencing data set of the dorsolateral prefrontal cortex obtained from autopsied, elderly individuals enrolled in the Religious Orders Study and Rush Memory and Aging Project joint cohorts. Samples were collected from the cohort study with enrollment from 1994 to 2017. Data were last analyzed in May 2021.

**Main Outcomes and Measures:**

The main analysis examined whether X chromosome gene expression measured by RNA sequencing of the dorsolateral prefrontal cortex was associated with cognitive change during aging and AD, independent of AD pathology and at the transcriptome-wide level in women and men. Whether X chromosome gene expression was associated with neurofibrillary tangle burden, a measure of tau pathology that influences cognition, in women and men was also explored.

**Results:**

Samples for RNA sequencing of the dorsolateral prefrontal cortex were obtained from 508 individuals (mean [SD] age at death, 88.4 [6.6] years; 315 [62.0%] were female; 197 [38.8%] had clinical diagnosis of AD at death; 293 [58.2%] had pathological diagnosis of AD at death) enrolled in the Religious Orders Study and Rush Memory and Aging Project joint cohorts and were followed up annually for a mean (SD) of 6.3 (3.9) years. X chromosome gene expression (29 genes), adjusted for age at death, education, and AD pathology, was significantly associated with cognitive change at the genome-wide level in women but not men. In the majority of identified X genes (19 genes), increased expression was associated with slower cognitive decline in women. In contrast with cognition, X chromosome gene expression (3 genes), adjusted for age at death and education, was associated with neuropathological tau burden at the genome-wide level in men but not women.

**Conclusions and Relevance:**

In this study, the X chromosome was associated with cognitive trajectories and neuropathological tau burden in aging and AD in a sex-specific manner. This is important because specific X chromosome factors could contribute risk or resilience to biological pathways of aging and AD in women, men, or both.

## Key Points

**Question:**

Is X chromosome gene expression in the brain associated with cognitive change or tau pathology in aging and Alzheimer disease in women and men?

**Findings:**

In this cohort study of 508 individuals, X chromosome gene expression assessed by RNA sequencing was associated with cognitive change in women but not men in a manner independent of Alzheimer disease pathology. In contrast with cognition, X chromosome gene expression was associated with neuropathologic tau burden in men but not women.

**Meaning:**

The X chromosome transcriptome, representing a significant portion of the genome of women and men, is associated with cognitive trajectories and neuropathological tau burden in aging and Alzheimer disease in a sex-specific manner.

## Teaser

This cohort study determines whether the X chromosome is associated with sex-specific cognitive change and tau pathology in aging and Alzheimer disease.

## Introduction

The X chromosome represents 5% of the genome in women and men and is understudied in aging and Alzheimer disease (AD). In the brain, more genes are expressed from the X chromosome than from any other single autosome^1^; however, analytic challenges posed by X hemizygosity in male individuals, random X inactivation and baseline X escape in female individuals, shared sequences between the X and Y, and limited representation of the X in genome-wide association studies, have largely led to its exclusion in studies.^2^ Despite historical constraints, tool kits are expanding, and varied sequencing approaches offer complimentary opportunities to investigate the X with high fidelity in brain aging and neurodegenerative disease. Any advances gained in dedicated study of the X are particularly important given its high density of neural genes, potential contribution to disease-relevant biology within and between each sex, and history of meaningful discoveries in other fields of medicine.^3,4^

With this in mind, we investigated an RNA sequencing (RNA-seq) data set from the well-established Religious Orders Study and Rush Memory and Aging Project joint cohorts to measure transcriptional levels of X gene expression in the dorsolateral prefrontal cortex, a cortical hub of multiple cognitive circuits, targeted by aging and AD.^5^ Because X gene expression is imbalanced between the sexes, we performed separate analyses of women and men. In our main analysis, we examined whether X expression is associated with cognitive change during aging and AD, independent of AD pathology, in women and men. We also explored whether X expression is associated with neurofibrillary tangle (NFT) burden, a major component of AD pathology linked with cognitive decline in women and men.

## Methods

We performed linear regressions of data derived from RNA-seq of dorsolateral prefrontal cortex with longitudinal change in global cognition and with NFT burden (assessed over 8 regions) in individuals from the Religious Orders Study and Rush Memory and Aging Project joint cohorts. Participants were without known dementia at enrollment (1994-2017) and were followed up longitudinally until death. Institutional review board approval was obtained from Rush University, and all participants provided written informed consent, agreed to brain donation, and signed a repository consent allowing their data to be repurposed. RNA-seq methods have been described in detail.^5^ Of 13 822 coding genes detected genome-wide, 488 were from the X chromosome. Neuropathological examination and antemortem clinical and neuropsychological profiling were performed.^5,6^ Global cognitive function was derived for each individual from the annual neuropsychological evaluation, comprising 17 different tests that were collapsed to form rates of cognitive decline, controlling for age and years of education.^5^ Cognitive decline was regressed against messenger RNA expression and covaried by extent of AD pathology (NFT and neuritic plaque scores); the association was defined as β. Brain NFT burden was regressed against messenger RNA expression, accounting for age and education; the association was defined as β. Additional methods are provided in the eMethods in the Supplement. Significance was established genome-wide at a false discovery rate–adjusted *P* value of less than .05. Analysis of women was performed separately from men owing to the biologic imbalance of X gene expression between the sexes. Analyses took place in May 2021.

## Results

Demographics of the samples from the Religious Orders Study and Rush Memory and Aging Project joint cohorts that underwent RNA-seq are shown in Table 1 and eTable 1 in the Supplement, with 508 individuals followed up longitudinally for a mean (SD) of 6.3 (3.9) years. Of these, 315 (62.0%) were female, 197 (38.8%) carried a clinical diagnosis of AD, and 296 (58.2%) carried a pathological diagnosis of AD. Most individuals (499 [98.2%]) self-reported as non-Hispanic White (eMethods in Supplement). Individuals with no cognitive impairment (166 [32.7%]), mild cognitive impairment (124 [24.4%]), clinical AD (173 [34.1%]), mixed mild cognitive impairment (9 [1.8%]), mixed AD (24 [4.7%]), and other dementias (12 [2.4%]) did not differ by sex (eTable 1 in the Supplement).

**Table 1.** Demographic Information for ROS/MAP Samples Used in RNA Sequencing of the Dorsolateral Prefrontal Cortex

Characteristic	ROS (n = 278)	MAP (n = 230)	ROS/MAP (N = 508)
Age, mean (SEM), y			
At enrollment	78.9 (7.1)	84.3 (5.8)	81.3 (7.0)
At death	87.6 (7.2)	89.3 (5.8)	88.4 (6.6)
Education, mean (SEM), y	18.0 (3.2)	14.7 (2.7)	16.5 (3.5)
Female, No. (%)	172 (61.8)	143 (62.2)	315 (62.0)
Male, No. (%)	106 (38.1)	87 (37.8)	193 (38.0)
Clinical diagnosis of AD at death, No. (%)	107 (38.5)	90 (39.1)	197 (38.8)
Pathological diagnosis of AD, No. (%)	162 (58.2)	134 (58.2)	296 (58.2)

Abbreviations: AD, Alzheimer disease; MAP, Rush Memory and Aging Project; ROS, Religious Orders Study.

In women, select X chromosome genes were significantly associated with cognitive change at the genome-wide level (29 genes) (Figure, A and B, Table 2, and eTable 2 in the Supplement), adjusted for age, education, and AD pathological burden. Of these, 19 genes (65.5%) showed a positive β score, indicating increased messenger RNA expression associated with slower cognitive decline. In men, X genes were not significantly associated with cognitive change (Table 2 and eTable 3 in the Supplement), despite similar cognitive decline to women (β = −0.18, *P* = .86; eTable 4 in the Supplement). While lower numbers of men contributed to decreased statistical power, subsampling of women to a male-equivalent–sized cohort continued to show significance of some genes, indicating female specificity of X chromosome–cognition associations. Nonetheless, β scores between women and men showed a strong statistical correlation revealing similar magnitude and direction of X expression with cognitive change between the sexes (Figure, C).

**Figure.**
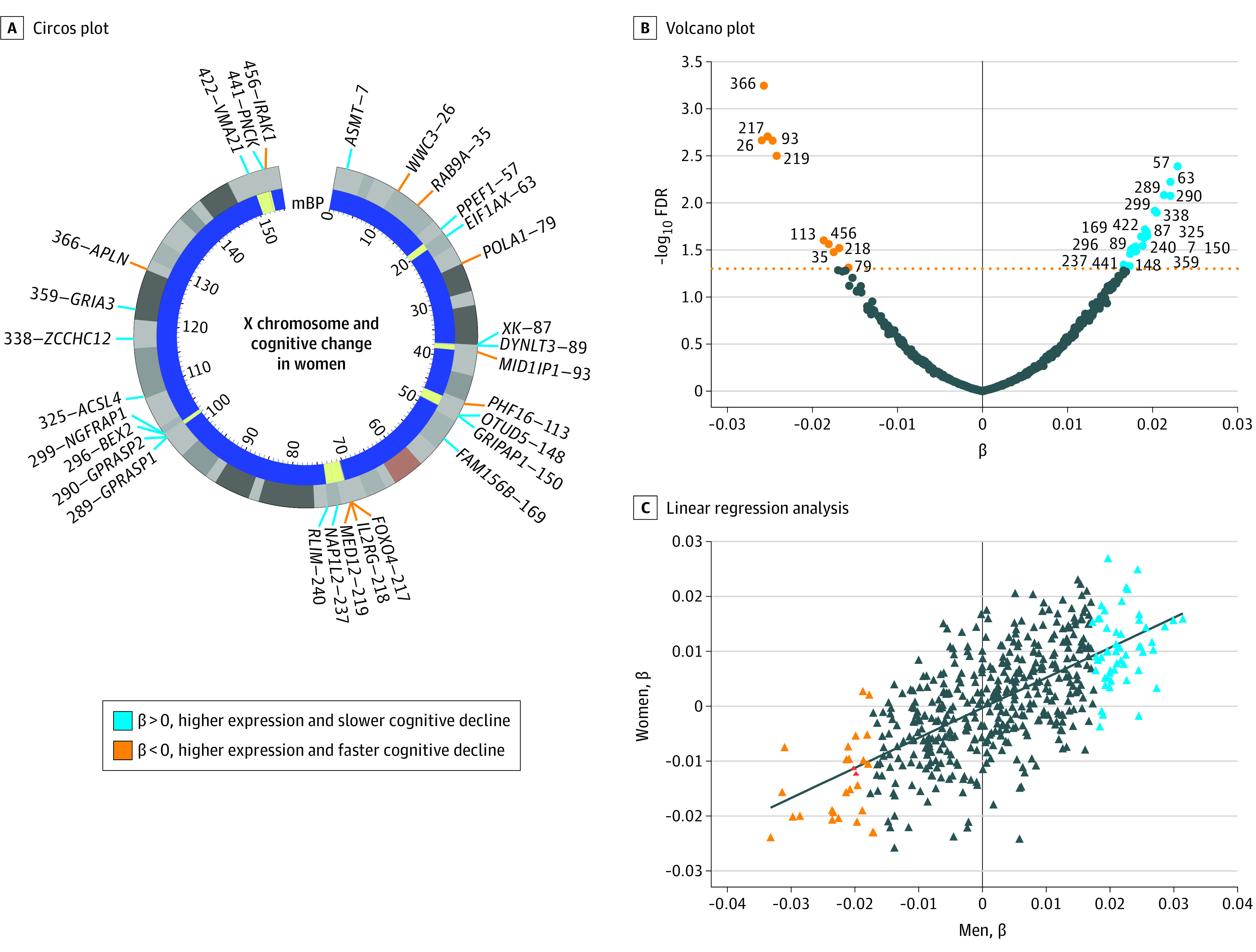
Association of X Chromosome Genes With Cognitive Change in Women in Aging and Alzheimer Disease A, Circos plot of the human X chromosome with coding genes that passed RNA sequencing threshold of significance after genome-wide correction in women, numbered consecutively by location. The inner dark blue band maps clusters of genes in yellow. B, Volcano plot shows β for each gene along with level of statistical significance. Numbering refers to location on X chromosome as depicted in panel A. C, Linear regression analysis of β scores are indicated between men and women (*P* < .001; *R^2^* = 0.33). FDR indicatesF false discovery rate; mBP, mega base-pairs.

**Table 2.** Association of X Chromosome Genes Expression With Cognition and Neurofibrillary Tangle Burden in a Sex-Specific Manner^a^

Characteristic	X-linked gene	Women		Men	
		β	*P* value	β	*P* value
Cognition	*APLN*	−0.0257	<.001^b^	−0.0141	.45
	*FOXO4*	−0.0253	.002^b^	−0.0166	.32
	*WWC3*	−0.0259	.002^b^	−0.0039	.86
	*MID1IP1*	−0.0247	.002^b^	−0.0144	.37
	*MED12*	−0.0242	.003^b^	−0.0143	.37
	*PPEF1*	0.0230	.004^b^	0.0157	.35
	*EIF1AX*	0.0221	.006^b^	0.0112	.54
	*GPRASP1*	0.0214	.008^b^	0.0124	.48
	*GPRASP2*	0.0221	.008^b^	0.0131	.41
	*NGFRAP1*	0.0203	.01^b^	0.0067	.75
	*ZCCHC12*	0.0205	.01^b^	0.0070	.74
	*FAM156B*	0.0191	.02^b^	0.0034	.90
	*XK*	0.0194	.02^b^	0.0096	.61
	*ACSL4*	0.0194	.02^b^	0.0033	.90
	*VMA21*	0.0187	.02^b^	0.0206	.21
	*RLIM*	0.0190	.02^b^	−0.0009	.98
	*PHF16*	−0.0187	.03^b^	−0.0096	.64
	*IRAK1*	−0.0181	.03^b^	−0.0100	.60
	*GRIPAP1*	0.0189	.03^b^	0.0038	.88
	*BEX2*	0.0180	.03^b^	0.0119	.51
	*IL2RG*	−0.0168	.03^b^	−0.0105	.62
	*ASMTL*	0.0177	.03^b^	0.0157	.36
	*NAP1L2*	0.0175	.03^b^	0.0088	.66
	*OTUD5*	0.0181	.03^b^	0.0083	.65
	*RAB9A*	−0.0175	.03^b^	−0.0141	.41
	*DYNLT3*	0.0174	.03^b^	0.0126	.49
	*PNCK*	0.0166	.045^b^	0.0058	.80
	*GRIA3*	0.0173	.047^b^	0.0098	.60
	*POLA1*	−0.0157	.049^b^	−0.0137	.47
Neurofibrillary tangles	*EMD*	−0.0325	.57	−0.0982	.03^b^
	*UBL4A*	0.0206	.75	0.0975	.03^b^
	*PHF16*	0.0343	.57	0.0901	.03^b^

^a^ Models for each sex include covariates for age at death and educational attainment.

^b^ Significant false discovery rate–adjusted *P* values for genome-wide correction are indicated.

In contrast with cognition, X chromosome gene expression was associated with NFT burden at the genome-wide level (3 genes) in men (Table 2; eTable 5 in the Supplement) but not women (Table 2 and eTable 6 in the Supplement). This is despite the lower NFT burden in men (β = −0.06, *P* = .07; eTable 4 in the Supplement) compared with women.

## Discussion

Significant associations of the X chromosome with cognitive change and tau pathology in aging and AD were sex specific. X chromosome gene expression assessed by RNA-seq in the dorsolateral prefrontal cortex was associated with cognitive change in women but not men, independent of AD pathology. In contrast with cognition, X gene expression was associated with neuropathological tau burden in men but not women.

Sex-specific findings of X gene expression in aging and AD were observed at the genome-wide level, including statistical correction for all autosomal and X genes detected. Thus, our results represent strong biological signals comparable with studies reporting autosomal gene associations. Sex stratification likely increased accuracy and resolution of findings because sex-specific biology governs X expression.

For the majority of identified X genes, higher levels were associated with slower cognitive decline in women. Among these, *GRIA3*, *GPRASP2*, and *GRIPAP1* (or *GRASP1*) code for proteins critical to mechanisms of synaptic transmission and plasticity, substrates of cognition. It is possible that women with a higher X dose from baseline escape or reactivation of the silent X showed resilience and better cognitive outcomes, compared with women with a lower X dose. Female-specific X biology, including harboring a second X chromosome, could also contribute to sex differences favoring female individuals.^7^ This includes female longevity in AD^7^ and female resilience to higher tau burden.^8,9^ Of note, more women have AD in large part owing to their longevity with the disease, along with survival to advanced ages when risk and incidence is highest.^10^ Causal biological studies of X factors are needed for a deeper understanding for any of these putative roles.

Our observation that X chromosome gene expression, like *UBL4A,* which encodes a protein folding factor, is associated with neuropathological tau burden in men but not women could represent male-specific X biology. Emerging sex differences in tau observed in human populations,^8,9^ along with increased tau-induced gene expression in male mice,^11^ support this possibility. Male-specific X biology includes hemizygosity of the X and maternal X inheritance, sources of genetic and epigenetic sex difference.

The spatial landscape of significant associations with cognitive change revealed transcriptional hot spots of genes clustered proximally on the X chromosome (Figure, A), suggesting common epigenetic regulators. Among these hot spots are genes linked to cognitive preservation and longevity protein families, including *MED12* and *FOXO4*. Whether they could contribute resilience or risk in aging and AD remains unknown.

Recent databases increasingly cover the X chromosome with high fidelity using varied informatic approaches, from expanded genome-wide association studies with X genetic variants^12^ to RNA-seq^13,14^ for direct gene expression levels, enabling proper X investigation. Two studies^13,14^ using single-cell RNA-seq broadly identified genes linked with AD phenotypes and detected X expression. One gene, *MID1IP1*, also emerged in our study. Its putative role modulating a phosphatase dysregulated in tau biology highlights how a deeper dive into X factors might reveal important pathways.

### Limitations

Limitations and caveats of our work include study of predominantly non-Hispanic White individuals within the United States, focus on 1 affected brain region, and lack of cell-type specificity of gene expression changes. It remains to be determined how broadly our findings extend and if X associations could differ with aging vs AD, not separated in this study.

## Conclusions

A disproportionate density of factors influencing neural function reside on the X chromosome^15^ and their roles in aging, AD, and other neurodegenerative diseases require identification and investigation in both sexes. This is important because X factors could contribute understanding of disease-relevant neurobiology along with sex differences and sex specificity of biomarkers, disease courses, and eventually pathways for personalized treatments against pathological aging and AD for women and men.

## Notes

Supplement. eMethods. eTable 1. Clinical Diagnoses of Patients in ROSMAP cohort separated by sex eTable 2. Association of X chromosome gene expression with cognition, adjusted for age, education, and AD pathology, in women eTable 3. Association of X chromosome gene expression with cognition, adjusted for age, education, and AD pathology, in men eTable 4. Global cognitive decline and NFT in men compared to women, adjusted by age and education eTable 5. Association of X chromosome gene expression with neurofibrillary tangles in women, adjusted by age and education eTable 6. Association of X chromosome gene expression with neurofibrillary tangles in men, adjusted by age and education eReferences.

## References

[1] Nguyen DK, Disteche CM. Dosage compensation of the active X chromosome in mammals. Nat Genet. 2006;38(1):47-53. doi:10.1038/ng1705 16341221

[2] Wise AL, Gyi L, Manolio TA. eXclusion: toward integrating the X chromosome in genome-wide association analyses. Am J Hum Genet. 2013;92(5):643-647. doi:10.1016/j.ajhg.2013.03.017 23643377 PMC3644627

[3] Sidorenko J, Kassam I, Kemper KE, . The effect of X-linked dosage compensation on complex trait variation. Nat Commun. 2019;10(1):3009. doi:10.1038/s41467-019-10598-y 31285442 PMC6614401

[4] Natarajan P, Pampana A, Graham SE, ; NHLBI Trans-Omics for Precision Medicine (TOPMed) Consortium; FinnGen. Chromosome Xq23 is associated with lower atherogenic lipid concentrations and favorable cardiometabolic indices. Nat Commun. 2021;12(1):2182. doi:10.1038/s41467-021-22339-1 33846329 PMC8042019

[5] Mostafavi S, Gaiteri C, Sullivan SE, . A molecular network of the aging human brain provides insights into the pathology and cognitive decline of Alzheimer’s disease. Nat Neurosci. 2018;21(6):811-819. doi:10.1038/s41593-018-0154-9 29802388 PMC6599633

[6] Bennett DA, Buchman AS, Boyle PA, Barnes LL, Wilson RS, Schneider JA. Religious Orders Study and rush memory and aging project. J Alzheimers Dis. 2018;64(s1):S161-S189. doi:10.3233/JAD-179939 29865057 PMC6380522

[7] Davis EJ, Broestl L, Abdulai-Saiku S, . A second X chromosome contributes to resilience in a mouse model of Alzheimer’s disease. Sci Transl Med. 2020;12(558):eaaz5677. doi:10.1126/scitranslmed.aaz5677 32848093 PMC8409261

[8] Ossenkoppele R, Lyoo CH, Jester-Broms J, . Assessment of demographic, genetic, and imaging variables associated with brain resilience and cognitive resilience to pathological tau in patients with Alzheimer disease. JAMA Neurol. 2020;77(5):632-642. doi:10.1001/jamaneurol.2019.5154 32091549 PMC7042808

[9] Dubal DB. Sex Difference in Alzheimer’s disease: an updated, balanced and emerging perspective on differing vulnerabilities. In: Lanzberger R, Kranz GS, Savic I, eds. Sex Differences in Neurology and Psychiatry. Elsevier; 2020. doi:10.1016/B978-0-444-64123-6.00018-7 33008530

[10] Shaw C, Hayes-Larson E, Glymour MM, . Evaluation of selective survival and sex/gender differences in dementia incidence using a simulation model. JAMA Netw Open. 2021;4(3):e211001. doi:10.1001/jamanetworkopen.2021.1001 33687445 PMC7944377

[11] Kodama L, Guzman E, Etchegaray JI, . Microglial microRNAs mediate sex-specific responses to tau pathology. Nat Neurosci. 2020;23(2):167-171. doi:10.1038/s41593-019-0560-7 31873194 PMC7394069

[12] Smith SM, Douaud G, Chen W, . An expanded set of genome-wide association studies of brain imaging phenotypes in UK Biobank. Nat Neurosci. 2021;24(5):737-745. doi:10.1038/s41593-021-00826-4 33875891 PMC7610742

[13] Grubman A, Chew G, Ouyang JF, . A single-cell atlas of entorhinal cortex from individuals with Alzheimer’s disease reveals cell-type-specific gene expression regulation. Nat Neurosci. 2019;22(12):2087-2097. doi:10.1038/s41593-019-0539-4 31768052

[14] Mathys H, Davila-Velderrain J, Peng Z, . Single-cell transcriptomic analysis of Alzheimer’s disease. Nature. 2019;570(7761):332-337. doi:10.1038/s41586-019-1195-2 31042697 PMC6865822

[15] Skuse DH. X-linked genes and mental functioning. Hum Mol Genet. 2005;14(Spec No 1):R27-R32. doi:10.1093/hmg/ddi112 15809269

