# Supplementary material for: Sex-Specific Association of the X Chromosome With Cognitive Change and Tau Pathology in Aging and Alzheimer Disease
Source: JAMA Neurol. 2021 Aug 23;78(10):1–6. doi: 10.1001/jamaneurol.2021.2806 (PMC8383157; doi:10.1001/jamaneurol.2021.2806)
Supplement: Supplement. — eMethods. eTable 1. Clinical Diagnoses of Patients in ROSMAP cohort separated by sex eTable 2. Association of X chromosome gene expression with cognition, adjusted for age, education, and AD pathology, in women eTable 3. Association of X chromosome gene expression with cognition, adjusted for age, education, and AD pathology, in men eTable 4. Global cognitive decline and NFT in men compared to women, adjusted by age and education eTable 5. Association of X chromosome gene expression with neurofibrillary tangles in women, adjusted by age and education eTable 6. Association of X chromosome gene expression with neurofibrillary tangles in men, adjusted by age and education eReferences. [file jamaneurol-e212806-s001.pdf]

Supplemental Online Content

Davis EJ, Solsberg CW, White CC, et al. Sex-specific association of the X chromosome with cognitive change and tau pathology in aging and Alzheimer disease. *JAMA Neurol*. Published online August 23, 2021. doi:10.1001/jamaneurol.2021.2806

eMethods.
eTable 1. Clinical Diagnoses of Patients in ROSMAP cohort separated by sex
eTable 2. Association of X chromosome gene expression with cognition, adjusted for age, education, and AD pathology, in women
eTable 3. Association of X chromosome gene expression with cognition, adjusted for age, education, and AD pathology, in men
eTable 4. Global cognitive decline and NFT in men compared to women, adjusted by age and education
eTable 5. Association of X chromosome gene expression with neurofibrillary tangles in women, adjusted by age and education
eTable 6. Association of X chromosome gene expression with neurofibrillary tangles in men, adjusted by age and education
eReferences.

This supplemental material has been provided by the authors to give readers additional information about their work.

© 2021 Davis EJ et al. *JAMA Neurology.*

eMethods

Participants. Participants were without known dementia at enrollment, agreed to brain donation, signed a repository consent allowing their data to be repurposed, and were longitudinally followed until death. Individuals were mostly of a non-Hispanic White background (311 of 315 women with 2 Hispanic White and 2 non-Hispanic Black backgrounds; 188 of 193 men with 2 Hispanic White and 3 non-Hispanic Black backgrounds).

RNA Sequencing. RNA-seq was carried out with the Illumina HiSeq2000 with 101 bp paired end reads for a targeted coverage of 50M paired reads. RNA-seq data quantification, quality control, data normalization, and association analyses have been described in detail[1] and include batch correction and removal of the effects of major biological (including age at death and sex) and technical (RIN, number of ribosomal bases, number of aligned reads, study index, and postmortem interval) confounding factors. Genes with at least 4 reads detected in at least 100 individuals were analyzed. The current analysis of coding genes yielded 13,822 genes including 488 X chromosome genes. In association analyses reported, significance was established genome-wide at a false discovery rate (FDR) adjusted p-value of <0.05. We performed linear regressions of data derived from RNA-seq of DLPFC with longitudinal change in global cognition and with NFT burden in individuals from the ROS/MAP joint cohorts (Table 1; eTable 1) using R (version 3.5, lm function).

Cognitive testing. The ROS and MAP methods of assessing cognition have been summarized in previous publications[1-4]. Briefly, uniform and comprehensive cognitive assessments were administered annually to the ROS and MAP participants; annual follow-up was similar between women and men (cohort-at-large, sampled 2012-2016, p=0.22 by chi-square). Scores from 17 cognitive performance tests common to both studies were used to obtain a summary measure for global cognition. Each test was converted to a z-score based on the mean and standard deviation of the scores from all participants at baseline. The z-scores were averaged. The mean global cognition is the mean of both cohorts together at baseline and each unit is roughly one standard deviation. To obtain a measurement of cognitive change, the annual global cognitive scores were modeled longitudinally with a mixed effects model, adjusting for age, sex and education, providing the rate of cognitive change for each person.

Tau Burden. Tau burden, via measurement of neurofibrillary tangle (NFT), for each individual was assessed across eight regions of the brain to create a single summary measure as previously described[1]. Using global tau burden is relevant to the DLPFC because global tau deposition causes dysfunction across multiple connected regions and networks. Since the DLPFC is a cognitive hub with inputs from multiple regions and networks, correlating global tau burden with RNA seq of this area facilitates an approach that assess both direct and indirect, proximal and distal, influences of tau pathology. Briefly, to quantify an estimate of the burden of abnormally phosphorylated tau-positive NFT (PHFtau) levels present in the cortex at death, tissue was dissected from eight regions of the brain: the hippocampus, entorhinal cortex, anterior cingulate cortex, midfrontal cortex, superior frontal cortex, inferior temporal cortex, angular gyrus and calcarine cortex. Sections 20 µm thick from each region were stained with antibodies to the tau protein and quantified with image analysis and stereology as previously described[5,6]. Briefly, PHFtau was labeled with the AT8 antibody. Using stereological mapping, the average density (per mm^2) of PHFtau tangles was determined and scores across the eight regions were averaged to create a single summary measure.

Clinical Diagnosis of AD at the time of death. The clinical diagnosis of AD dementia follows the recommendation of the joint working group of the National Institute of Neurological and Communicative Disorders and Stroke and the AD and Related Disorders Association[7] and was previously described[1]. Briefly, the diagnosis required a history of cognitive decline along with impairment in memory and at least one other cognitive domain. After a participant died, a neurologist specializing in dementia reviewed clinical information and provided a summary

© 2021 Davis EJ et al. *JAMA Neurology.*

opinion of the most likely clinical diagnosis at the time of death. This summary diagnosis was blinded to all neuropathologic data; case conferences were held for consensus as necessary. AD dementia includes persons with probable or possible AD dementia.

Sex differences in global cognitive change and NFT burden. Sex differences in each of these measures was measured by linear regression, adjusted by age at death and education.

© 2021 Davis EJ et al. *JAMA Neurology.*

eReferences

1. Mostafavi S, Gaiteri C, Sullivan SE, et al. A molecular network of the aging human brain provides insights into the pathology and cognitive decline of Alzheimer's disease. *Nat Neurosci.* 2018;21(6):811-819.
2. Bennett DA, Schneider JA, Arvanitakis Z, et al. Neuropathology of older persons without cognitive impairment from two community-based studies. *Neurology.* 2006;66(12):1837-1844.
3. Wilson RS, Beckett LA, Barnes LL, et al. Individual differences in rates of change in cognitive abilities of older persons. *Psychol Aging.* 2002;17(2):179-193.
4. Wilson RS, Boyle PA, Yu L, Segawa E, Sytsma J, Bennett DA. Conscientiousness, dementia related pathology, and trajectories of cognitive aging. *Psychol Aging.* 2015;30(1):74-82.
5. Bennett DA, Wilson RS, Boyle PA, Buchman AS, Schneider JA. Relation of neuropathology to cognition in persons without cognitive impairment. *Ann Neurol.* 2012;72(4):599-609.
6. Boyle PA, Wilson RS, Yu L, et al. Much of late life cognitive decline is not due to common neurodegenerative pathologies. *Ann Neurol.* 2013;74(3):478-489.
7. McKhann G, Drachman D, Folstein M, Katzman R, Price D, Stadlan EM. Clinical diagnosis of Alzheimer's disease: report of the NINCDS-ADRDA Work Group under the auspices of Department of Health and Human Services Task Force on Alzheimer's Disease. *Neurology*. 1984;34(7):939-944.

© 2021 Davis EJ et al. *JAMA Neurology.*

eTable 1. Clinical Diagnoses of Patients in ROSMAP cohort separated by sex			
	**Women (n=315)†**	**Men (n=193)†**	**Total (n=508)**
NCI	98 (31.1%)	68 (35.2%)	166 (32.7%)
MCI no other cause	79 (25.1%)	45 (23.3%)	124 (24.4%)
MCI plus other cause	3 (1.0%)	6 (3.1%)	9 (1.8%)
AD and no other cause	118 (37.5%)	55 (28.5%)	173 (34.1%)
AD plus other cause	11 (3.5%)	13 (6.7%)	24 (4.7%)
Other dementia	6 (1.9%)	6 (3.1%)	12 (2.4%)

Data shown as count (percent of column total).

NCI=no cognitive impairment; MCI=mild cognitive impairment; AD=Alzheimer's disease

† women and men diagnostic distributions do not differ significantly (chi-square test p-value=0.24)

© 2021 Davis EJ et al. *JAMA Neurology.*

**eTable 2. Association of X chromosome gene expression with cognition, adjusted for age, education, and AD pathology, in women**. FDR-adjusted p-values for genome-wide correction shown.			
**Gene**	**Beta**	**Pvalue**	**Pvalue Adjusted**
APLN	-0.0257	0.0000	**0.0006**
FOXO4	-0.0253	0.0000	**0.0020**
WWC3	-0.0259	0.0000	**0.0022**
MID1IP1	-0.0247	0.0000	**0.0022**
MED12	-0.0242	0.0000	**0.0032**
PPEF1	0.0230	0.0000	**0.0041**
EIF1AX	0.0221	0.0000	**0.0060**
GPRASP1	0.0214	0.0000	**0.0082**
GPRASP2	0.0221	0.0000	**0.0084**
NGFRAP1	0.0203	0.0001	**0.0121**
ZCCHC12	0.0205	0.0001	**0.0127**
FAM156B	0.0191	0.0002	**0.0191**
XK	0.0194	0.0002	**0.0204**
ACSL4	0.0194	0.0002	**0.0224**
VMA21	0.0187	0.0002	**0.0228**
RLIM	0.0190	0.0003	**0.0235**
PHF16	-0.0187	0.0003	**0.0250**
IRAK1	-0.0181	0.0004	**0.0274**
GRIPAP1	0.0189	0.0005	**0.0285**
BEX2	0.0180	0.0005	**0.0290**
IL2RG	-0.0168	0.0005	**0.0301**
ASMTL	0.0177	0.0005	**0.0303**
NAP1L2	0.0175	0.0006	**0.0312**
OTUD5	0.0181	0.0006	**0.0326**
RAB9A	-0.0175	0.0006	**0.0333**
DYNLT3	0.0174	0.0007	**0.0347**
PNCK	0.0166	0.0011	**0.0453**
GRIA3	0.0173	0.0012	**0.0468**
POLA1	-0.0157	0.0013	**0.0487**
PRKX	-0.0170	0.0015	0.0518
BRCC3	0.0166	0.0015	0.0522
HMGN5	-0.0161	0.0016	0.0524
ENOX2	-0.0164	0.0016	0.0535
HS6ST2	0.0169	0.0017	0.0537
KCND1	0.0167	0.0017	0.0543
STS	0.0166	0.0019	0.0572
DLG3	0.0160	0.0020	0.0596
ATP2B3	0.0161	0.0022	0.0621
CCDC160	-0.0153	0.0022	0.0624
FAM127A	0.0161	0.0023	0.0629
FRMPD4	0.0160	0.0025	0.0654
PIM2	0.0156	0.0027	0.0685
RRAGB	0.0155	0.0027	0.0686
CXorf40A	0.0158	0.0029	0.0717
ARHGEF9	0.0157	0.0030	0.0720
PRPS2	0.0153	0.0030	0.0724

© 2021 Davis EJ et al. *JAMA Neurology.*

SLC25A14	0.0152	0.0031	0.0734
SYP	0.0150	0.0033	0.0752
ABCD1	-0.0157	0.0035	0.0759
ARHGEF6	-0.0143	0.0035	0.0764
PJA1	0.0147	0.0036	0.0770
HPRT1	0.0148	0.0037	0.0785
NKRF	0.0149	0.0038	0.0795
UTP14A	0.0153	0.0039	0.0804
REPS2	0.0150	0.0043	0.0845
ZFX	-0.0147	0.0043	0.0853
RNF128	0.0145	0.0045	0.0872
RENBP	-0.0148	0.0045	0.0875
PRICKLE3	-0.0142	0.0047	0.0892
TCEAL6	0.0144	0.0048	0.0903
DACH2	0.0140	0.0057	0.0980
ATP6AP2	0.0142	0.0061	0.1020
ATP6AP1	0.0145	0.0063	0.1034
CETN2	0.0139	0.0071	0.1084
SMC1A	-0.0129	0.0075	0.1117
CXorf40B	0.0139	0.0076	0.1123
MCTS1	0.0135	0.0078	0.1133
MAGED1	0.0135	0.0079	0.1138
ARMCX3	0.0139	0.0080	0.1145
TIMM8A	0.0147	0.0082	0.1165
SLITRK4	0.0139	0.0086	0.1186
TAF9B	0.0133	0.0086	0.1188
GDI1	0.0130	0.0087	0.1196
CD99L2	0.0139	0.0088	0.1203
DDX26B	0.0133	0.0094	0.1247
MAGEH1	0.0136	0.0096	0.1255
TSPAN6	-0.0135	0.0096	0.1256
ZRSR2	-0.0134	0.0100	0.1288
MED14	0.0134	0.0105	0.1313
TXLNG	0.0138	0.0108	0.1324
ELK1	-0.0132	0.0111	0.1344
NXT2	-0.0131	0.0113	0.1354
STAG2	-0.0135	0.0116	0.1368
GPM6B	-0.0129	0.0116	0.1372
PABPC1L2A	0.0130	0.0120	0.1388
UBA1	-0.0127	0.0121	0.1393
ARMCX5	0.0131	0.0127	0.1424
PHF8	-0.0127	0.0130	0.1441
UXT	-0.0131	0.0133	0.1458
PSMD10	0.0121	0.0141	0.1505
ARMCX2	0.0126	0.0141	0.1505
PNMA5	0.0125	0.0142	0.1507
PQBP1	-0.0131	0.0147	0.1526
UPRT	0.0126	0.0147	0.1527
PNMA3	0.0125	0.0148	0.1531

© 2021 Davis EJ et al. *JAMA Neurology.*

MBTPS2	0.0130	0.0156	0.1559
RAB9B	0.0128	0.0156	0.1560
CDKL5	0.0127	0.0161	0.1581
ZBED1	-0.0122	0.0164	0.1596
ATP11C	-0.0119	0.0165	0.1601
PRRG3	0.0128	0.0170	0.1616
HCFC1	-0.0120	0.0177	0.1651
PGRMC1	0.0126	0.0185	0.1678
LAMP2	-0.0120	0.0186	0.1680
TCEAL7	0.0125	0.0189	0.1693
TMEM35	0.0119	0.0190	0.1700
RBM10	0.0122	0.0195	0.1724
DRP2	0.0122	0.0197	0.1734
MUM1L1	0.0121	0.0199	0.1740
ERAS	0.0118	0.0203	0.1753
TMSB4X	0.0115	0.0204	0.1759
ZC4H2	0.0126	0.0209	0.1781
SLC25A53	-0.0119	0.0214	0.1801
BEX5	0.0115	0.0217	0.1813
BCAP31	0.0118	0.0220	0.1827
AMOT	-0.0116	0.0224	0.1847
SYTL4	-0.0115	0.0238	0.1902
EIF2S3	0.0119	0.0240	0.1911
GABRA3	0.0116	0.0243	0.1924
EMD	0.0114	0.0247	0.1934
PRRG1	-0.0116	0.0262	0.1996
NLGN3	-0.0111	0.0263	0.2000
USP11	0.0114	0.0264	0.2002
XIAP	0.0113	0.0267	0.2016
AP1S2	0.0113	0.0270	0.2024
SMS	0.0115	0.0275	0.2042
MECP2	-0.0115	0.0278	0.2047
PORCN	0.0115	0.0287	0.2078
MPP1	0.0114	0.0290	0.2093
TCEAL5	0.0109	0.0295	0.2109
MAGEE1	0.0111	0.0300	0.2131
THOC2	-0.0109	0.0307	0.2156
PABPC1L2B	0.0112	0.0317	0.2178
ZNF275	-0.0109	0.0323	0.2199
NUDT11	0.0108	0.0331	0.2229
MORF4L2	0.0109	0.0340	0.2258
MTM1	-0.0103	0.0342	0.2264
FGF13	0.0109	0.0354	0.2300
LONRF3	-0.0110	0.0356	0.2306
BEX1	0.0108	0.0358	0.2311
PDK3	0.0109	0.0377	0.2367
TBL1X	-0.0104	0.0378	0.2372
BEX4	0.0107	0.0382	0.2388
PRPS1	0.0107	0.0396	0.2437

© 2021 Davis EJ et al. *JAMA Neurology.*

HNRNPH2	0.0107	0.0398	0.2441
AKAP17A	-0.0108	0.0399	0.2444
WBP5	-0.0107	0.0401	0.2453
MCF2	0.0105	0.0410	0.2480
CXorf24	-0.0111	0.0411	0.2483
F8	0.0106	0.0427	0.2535
TAB3	0.0109	0.0430	0.2543
GPR34	-0.0100	0.0466	0.2642
FAAH2	0.0100	0.0473	0.2663
KLF8	0.0098	0.0485	0.2696
SHROOM2	0.0105	0.0487	0.2701
NAP1L3	0.0100	0.0523	0.2798
RAB39B	0.0101	0.0526	0.2805
STARD8	-0.0099	0.0526	0.2806
GPC4	-0.0095	0.0546	0.2862
ZNF75D	0.0101	0.0552	0.2884
PPP1R3F	0.0096	0.0553	0.2885
HSD17B10	-0.0098	0.0557	0.2886
GLA	0.0098	0.0559	0.2891
ZMAT1	0.0098	0.0561	0.2896
HMGB3	0.0100	0.0564	0.2905
TSR2	0.0098	0.0580	0.2949
WDR13	-0.0093	0.0597	0.2992
IDS	0.0099	0.0598	0.2994
SLC9A6	0.0100	0.0599	0.2997
SPIN2B	0.0096	0.0605	0.3013
PTCHD1	-0.0097	0.0635	0.3077
ARAF	-0.0093	0.0678	0.3169
MAGED4B	-0.0096	0.0679	0.3171
PLP1	-0.0095	0.0682	0.3178
BHLHB9	0.0094	0.0696	0.3210
CXorf36	-0.0093	0.0711	0.3243
PIGA	-0.0093	0.0719	0.3263
AMMECR1	0.0092	0.0732	0.3293
UBQLN2	0.0095	0.0748	0.3321
OCRL	0.0093	0.0768	0.3361
KIAA2022	0.0091	0.0784	0.3406
PLXNB3	-0.0091	0.0794	0.3422
IL3RA	-0.0090	0.0802	0.3440
TSPAN7	0.0093	0.0804	0.3444
TMEM47	-0.0089	0.0828	0.3482
DCX	0.0086	0.0873	0.3589
NLGN4X	0.0091	0.0875	0.3593
NUDT10	0.0088	0.0876	0.3595
SASH3	-0.0084	0.0881	0.3604
PPP2R3B	-0.0087	0.0884	0.3610
GSPT2	0.0090	0.0929	0.3700
PGK1	0.0085	0.0936	0.3712
FAM199X	0.0088	0.0947	0.3728

© 2021 Davis EJ et al. *JAMA Neurology.*

DHRSX	-0.0085	0.0949	0.3729
SHROOM4	-0.0087	0.0970	0.3767
ZXDB	0.0086	0.0972	0.3771
TBC1D8B	-0.0088	0.0974	0.3776
ALG13	0.0085	0.0989	0.3798
FAM123B	0.0082	0.1058	0.3932
SLC9A7	0.0087	0.1080	0.3972
APOO	-0.0080	0.1098	0.4007
TIMM17B	0.0079	0.1119	0.4042
HUWE1	-0.0080	0.1121	0.4047
SLC35A2	0.0082	0.1141	0.4078
MAP7D2	0.0081	0.1142	0.4080
HEPH	-0.0080	0.1152	0.4099
DMRTC1B	0.0080	0.1156	0.4107
TCEAL2	0.0080	0.1171	0.4122
WAS	-0.0082	0.1168	0.4122
F8A2	-0.0084	0.1175	0.4128
RP11-274K13.2	-0.0082	0.1189	0.4159
CCDC120	-0.0079	0.1193	0.4163
F8A3	-0.0083	0.1200	0.4173
FAM70A	-0.0080	0.1226	0.4214
TRO	0.0079	0.1231	0.4224
CITED1	0.0077	0.1236	0.4230
RNF113A	-0.0082	0.1247	0.4250
ZNF711	0.0081	0.1253	0.4256
CUL4B	0.0078	0.1258	0.4262
SEPT	0.0080	0.1269	0.4278
GJB1	-0.0081	0.1275	0.4288
USP51	0.0075	0.1275	0.4288
GTPBP6	-0.0078	0.1279	0.4294
EBP	0.0077	0.1286	0.4304
FMR1	0.0078	0.1289	0.4310
TAZ	-0.0079	0.1312	0.4343
PIR	0.0082	0.1321	0.4358
SLITRK2	-0.0076	0.1332	0.4370
RGAG4	0.0077	0.1348	0.4399
MTCP1NB	-0.0075	0.1417	0.4514
RAB33A	-0.0078	0.1437	0.4539
RPL36A	-0.0076	0.1461	0.4573
ZNF673	0.0077	0.1477	0.4593
CNKSR2	0.0076	0.1483	0.4599
FAM120C	-0.0071	0.1521	0.4646
FAM127C	-0.0077	0.1524	0.4649
HAUS7	0.0072	0.1535	0.4661
YIPF6	-0.0072	0.1544	0.4677
RP1-177G6.2	-0.0073	0.1552	0.4686
EFHC2	-0.0071	0.1600	0.4753
SLC25A43	-0.0075	0.1623	0.4780
CXorf38	-0.0069	0.1637	0.4799

© 2021 Davis EJ et al. *JAMA Neurology.*

ZC3H12B	0.0072	0.1639	0.4803
KDM6A	-0.0068	0.1653	0.4821
WASH6P	-0.0073	0.1659	0.4826
KLHL15	0.0071	0.1708	0.4896
RBM41	-0.0070	0.1712	0.4904
RBM3	0.0070	0.1729	0.4930
TCEAL1	-0.0072	0.1729	0.4930
CXorf26	0.0070	0.1808	0.5034
BRWD3	-0.0069	0.1842	0.5082
AL158821.1	-0.0070	0.1846	0.5087
PLS3	0.0068	0.1855	0.5099
SSR4	0.0072	0.1893	0.5144
RGN	-0.0068	0.1893	0.5145
DMD	-0.0068	0.1916	0.5166
DDX3X	-0.0064	0.1917	0.5168
NHS	-0.0067	0.1925	0.5179
RIBC1	0.0067	0.1944	0.5199
CSAG1	-0.0068	0.1966	0.5228
IGBP1	0.0068	0.1966	0.5228
TMSB15B	0.0066	0.2060	0.5343
SPIN3	0.0067	0.2067	0.5352
MSN	-0.0062	0.2083	0.5367
PNMA6C	0.0072	0.2103	0.5395
FAM50A	-0.0065	0.2109	0.5403
IL1RAPL1	0.0061	0.2139	0.5441
PNPLA4	0.0063	0.2146	0.5452
VBP1	0.0067	0.2159	0.5467
TCEAL8	0.0064	0.2175	0.5487
DIAPH2	0.0066	0.2206	0.5525
PRAF2	0.0063	0.2208	0.5528
L1CAM	0.0062	0.2216	0.5536
TMEM164	-0.0062	0.2276	0.5606
CLIC2	-0.0060	0.2370	0.5702
ARMCX1	0.0060	0.2372	0.5704
VSIG4	-0.0059	0.2381	0.5713
ZNF185	0.0063	0.2390	0.5724
MORC4	-0.0059	0.2469	0.5808
PHKA1	-0.0060	0.2485	0.5825
ARMCX6	-0.0058	0.2538	0.5877
NAA10	0.0059	0.2569	0.5909
FATE1	-0.0062	0.2572	0.5909
LDOC1	0.0060	0.2572	0.5909
SAT1	-0.0059	0.2571	0.5909
MAGIX	0.0057	0.2581	0.5918
WDR45	-0.0057	0.2614	0.5951
BTK	0.0056	0.2622	0.5960
CCDC22	0.0059	0.2646	0.5982
FTSJ1	0.0055	0.2659	0.5995
CHM	0.0058	0.2668	0.6003

© 2021 Davis EJ et al. *JAMA Neurology.*

CTPS2	0.0054	0.2822	0.6154
GK	0.0055	0.2846	0.6176
UBL4A	-0.0053	0.2859	0.6189
CHIC1	0.0055	0.2865	0.6196
KDM5C	-0.0052	0.2880	0.6212
CD99	-0.0055	0.2886	0.6217
PDZD11	0.0056	0.2888	0.6220
MMGT1	0.0055	0.2894	0.6227
CXorf69	-0.0053	0.2904	0.6237
MID1	-0.0052	0.2918	0.6245
PAK3	0.0052	0.2943	0.6263
ZMYM3	-0.0053	0.2968	0.6284
COX7B	0.0056	0.3037	0.6335
BGN	-0.0054	0.3043	0.6342
NONO	-0.0050	0.3103	0.6399
TSC22D3	-0.0052	0.3150	0.6444
PDZD4	0.0052	0.3182	0.6475
LAGE3	0.0051	0.3193	0.6485
KCNE1L	-0.0057	0.3209	0.6500
CDK16	-0.0050	0.3217	0.6505
GPR143	0.0051	0.3227	0.6516
IQSEC2	0.0050	0.3240	0.6527
PLP2	-0.0051	0.3242	0.6529
BCOR	0.0050	0.3253	0.6537
RAI2	-0.0050	0.3263	0.6541
COL4A5	-0.0050	0.3267	0.6543
PHF6	-0.0049	0.3292	0.6568
IL13RA1	-0.0050	0.3307	0.6580
TMEM185A	0.0050	0.3328	0.6599
F8A1	0.0053	0.3415	0.6668
BCORL1	-0.0047	0.3478	0.6713
EFNB1	-0.0050	0.3484	0.6714
SH3BGRL	-0.0046	0.3499	0.6728
ODZ1	0.0048	0.3509	0.6738
SPIN2A	0.0048	0.3554	0.6773
NDP	-0.0046	0.3594	0.6803
SLC6A8	-0.0047	0.3595	0.6803
IDH3G	-0.0046	0.3678	0.6870
FAM122C	0.0044	0.3691	0.6882
PDHA1	0.0049	0.3751	0.6927
ZNF630	0.0046	0.3766	0.6940
HCCS	0.0045	0.3835	0.6995
ARSE	-0.0045	0.3847	0.7007
TRMT2B	-0.0047	0.3867	0.7017
GNL3L	-0.0045	0.3909	0.7054
PIN4	-0.0045	0.3931	0.7069
SMPX	0.0044	0.3998	0.7120
CSF2RA	-0.0045	0.4003	0.7123
PLXNA3	0.0046	0.4005	0.7123

© 2021 Davis EJ et al. *JAMA Neurology.*

UPF3B	-0.0041	0.4091	0.7191
MSL3	0.0042	0.4154	0.7243
GDPD2	0.0041	0.4179	0.7256
MAMLD1	-0.0041	0.4194	0.7267
ACRC	-0.0040	0.4212	0.7277
MAOA	-0.0040	0.4316	0.7352
SCML1	-0.0041	0.4339	0.7369
MTCP1	0.0039	0.4346	0.7372
KLHL4	-0.0041	0.4369	0.7386
TRAPPC2	0.0040	0.4375	0.7389
FGD1	-0.0040	0.4410	0.7413
SRPK3	-0.0040	0.4452	0.7447
TBC1D25	0.0040	0.4465	0.7458
DUSP9	-0.0038	0.4472	0.7460
SYAP1	0.0039	0.4489	0.7471
ZNF41	-0.0040	0.4490	0.7472
TFE3	-0.0038	0.4495	0.7476
FAM122B	0.0041	0.4533	0.7505
PCDH11X	0.0040	0.4536	0.7505
CXorf1	0.0039	0.4549	0.7516
HDAC6	-0.0038	0.4585	0.7544
G6PD	-0.0039	0.4593	0.7546
GEMIN8	0.0037	0.4631	0.7567
CXorf56	0.0038	0.4646	0.7578
SYN1	0.0038	0.4692	0.7605
ZNF449	0.0039	0.4712	0.7623
PRDX4	0.0036	0.4716	0.7627
ZBTB33	-0.0037	0.4781	0.7669
IKBKG	0.0037	0.4795	0.7676
LAS1L	0.0035	0.4902	0.7727
FUNDC1	0.0035	0.4916	0.7732
MAP7D3	-0.0036	0.4950	0.7758
FAM156A	0.0036	0.4951	0.7759
RPL10	0.0034	0.5032	0.7804
HSFX1	-0.0033	0.5117	0.7860
TIMP1	0.0033	0.5149	0.7879
GPKOW	0.0031	0.5215	0.7916
DKC1	-0.0033	0.5225	0.7925
CHST7	-0.0033	0.5306	0.7968
FRMPD3	0.0033	0.5304	0.7968
WDR44	0.0032	0.5325	0.7978
OPHN1	-0.0032	0.5331	0.7984
CFP	0.0032	0.5374	0.8012
SMARCA1	-0.0031	0.5400	0.8027
SH3KBP1	0.0034	0.5422	0.8043
TSPYL2	0.0032	0.5456	0.8064
INE1	-0.0030	0.5481	0.8081
SLC38A5	-0.0029	0.5624	0.8154
GPR173	-0.0029	0.5626	0.8155

© 2021 Davis EJ et al. *JAMA Neurology.*

SNX12	0.0030	0.5677	0.8186
CASK	0.0029	0.5796	0.8249
FAM127B	-0.0028	0.5934	0.8342
PCDH19	0.0026	0.5935	0.8342
CA5B	-0.0028	0.5938	0.8343
MAGEE2	0.0028	0.5954	0.8348
ZNF674	-0.0027	0.5999	0.8368
PLCXD1	-0.0026	0.6059	0.8401
FAM155B	-0.0027	0.6080	0.8401
IGSF1	0.0027	0.6085	0.8401
UBE2A	0.0026	0.6097	0.8401
RPL39	0.0026	0.6135	0.8406
RPGR	-0.0026	0.6193	0.8428
CYSLTR1	-0.0025	0.6244	0.8456
FAM133A	0.0023	0.6335	0.8501
ATRX	0.0024	0.6391	0.8526
CSTF2	0.0025	0.6393	0.8527
FAM58A	-0.0023	0.6458	0.8559
CHRDL1	-0.0023	0.6510	0.8583
RP11-706O15.1	0.0022	0.6510	0.8583
ABCB7	-0.0022	0.6556	0.8605
RBBP7	-0.0023	0.6631	0.8647
CLCN4	0.0023	0.6644	0.8658
HTATSF1	-0.0022	0.6751	0.8707
RHOXF1	0.0024	0.6795	0.8724
ACOT9	-0.0021	0.6821	0.8739
RBMX	0.0022	0.6831	0.8744
CYBB	-0.0019	0.6937	0.8807
TCEAL3	-0.0020	0.6943	0.8810
MAOB	0.0019	0.6953	0.8816
ZNF182	0.0020	0.7026	0.8847
MID2	-0.0019	0.7033	0.8849
FAM3A	0.0019	0.7101	0.8884
MAGT1	0.0019	0.7130	0.8895
MOSPD1	-0.0019	0.7146	0.8900
FHL1	0.0017	0.7241	0.8946
PCSK1N	0.0017	0.7310	0.8981
ITM2A	0.0017	0.7349	0.9003
MAGED2	-0.0017	0.7384	0.9016
ARX	-0.0017	0.7439	0.9041
ARSD	0.0017	0.7447	0.9047
ZDHHC15	0.0017	0.7467	0.9056
VAMP7	0.0016	0.7518	0.9078
HDAC8	0.0016	0.7541	0.9089
KLHL13	-0.0016	0.7543	0.9090
FIGF	0.0016	0.7553	0.9094
CXorf57	0.0015	0.7734	0.9171
MTMR1	0.0014	0.7782	0.9192
LRCH2	0.0015	0.7834	0.9215

© 2021 Davis EJ et al. *JAMA Neurology.*

AFF2	0.0014	0.7900	0.9242
OGT	0.0013	0.7981	0.9279
SLC25A5	-0.0013	0.7999	0.9292
DNASE1L1	-0.0014	0.8006	0.9295
TMLHE	0.0012	0.8192	0.9364
NDUFB11	0.0011	0.8270	0.9399
TCEANC	-0.0012	0.8296	0.9407
TMEM187	-0.0011	0.8314	0.9414
TCEAL4	0.0011	0.8352	0.9426
ARSF	0.0010	0.8389	0.9439
ATG4A	-0.0010	0.8431	0.9449
ARHGAP4	-0.0010	0.8446	0.9453
PNMA6A	0.0010	0.8465	0.9460
FLNA	-0.0010	0.8539	0.9492
SRPX	-0.0010	0.8540	0.9492
FAM104B	0.0009	0.8552	0.9498
MAGEC3	0.0009	0.8664	0.9540
OFD1	-0.0009	0.8665	0.9540
NSDHL	0.0008	0.8751	0.9569
CXorf23	-0.0008	0.8766	0.9574
APOOL	-0.0008	0.8781	0.9581
SLC16A2	-0.0007	0.8849	0.9604
APEX2	-0.0007	0.8952	0.9640
RBMX2	0.0006	0.9062	0.9670
SLC25A6	0.0006	0.9073	0.9672
ZDHHC9	-0.0006	0.9078	0.9675
NDUFA1	-0.0006	0.9081	0.9676
ARMCX4	-0.0006	0.9102	0.9684
HDHD1	-0.0006	0.9120	0.9695
RPS6KA3	-0.0005	0.9192	0.9724
ZNF81	-0.0005	0.9232	0.9741
NKAP	0.0004	0.9395	0.9793
USP9X	0.0004	0.9425	0.9804
GYG2	0.0003	0.9451	0.9815
TAF1	-0.0003	0.9494	0.9829
MOSPD2	-0.0003	0.9531	0.9841
SLC10A3	-0.0003	0.9553	0.9848
RPS4X	0.0003	0.9601	0.9861
C1GALT1C1	0.0002	0.9607	0.9864
FUNDC2	0.0002	0.9632	0.9870
SCML2	0.0002	0.9657	0.9879
KAL1	-0.0002	0.9681	0.9888
RAP2C	-0.0001	0.9798	0.9935
AIFM1	0.0001	0.9808	0.9936
SUV39H1	0.0001	0.9837	0.9949
PCYT1B	0.0001	0.9850	0.9952
DMRTC1	0.0001	0.9912	0.9974
PHKA2	0.0001	0.9923	0.9979
MAGED4	0.0000	0.9933	0.9981

© 2021 Davis EJ et al. *JAMA Neurology.*

GABRE	0.0000	0.9939	0.9983

© 2021 Davis EJ et al. *JAMA Neurology.*

**eTable 3. Association of X chromosome gene expression with cognition, adjusted for age, education, and AD pathology, in men.** FDR-adjusted p-values for genome-wide correction shown.			
**Gene**	**Beta**	**Pvalue**	**Pvalue Adjusted**
TAF9B	0.0232	0.0013	0.1768
PTCHD1	-0.0218	0.0014	0.1805
FRMPD3	-0.0203	0.0027	0.2053
VMA21	0.0206	0.0035	0.2085
CLIC2	-0.0206	0.0036	0.2085
DNASE1L1	0.0182	0.0040	0.2150
WBP5	-0.0193	0.0047	0.2289
WASH6P	-0.0188	0.0072	0.2565
PRKX	-0.0174	0.0072	0.2565
TAZ	-0.0172	0.0105	0.2788
MECP2	-0.0167	0.0126	0.2922
ELK1	-0.0173	0.0129	0.2951
MAGED4B	-0.0171	0.0139	0.2998
BRCC3	0.0163	0.0142	0.3029
FTSJ1	-0.0176	0.0148	0.3066
TSC22D3	-0.0172	0.0152	0.3095
PDZD11	0.0165	0.0157	0.3121
FOXO4	-0.0166	0.0162	0.3160
HCFC1	-0.0170	0.0170	0.3205
TSR2	0.0161	0.0188	0.3282
FAM127A	0.0155	0.0196	0.3316
DMD	0.0153	0.0217	0.3401
MORC4	-0.0155	0.0229	0.3456
CSTF2	0.0153	0.0240	0.3499
PPEF1	0.0157	0.0242	0.3513
PRICKLE3	-0.0156	0.0249	0.3543
ASMTL	0.0157	0.0260	0.3589
RP11-706O15.1	0.0161	0.0265	0.3599
CXorf56	0.0147	0.0272	0.3627
HMGB3	0.0151	0.0284	0.3678
MID1IP1	-0.0144	0.0286	0.3688
MED12	-0.0143	0.0293	0.3714
PLS3	0.0152	0.0294	0.3715
PNMA5	0.0157	0.0308	0.3768
ITM2A	0.0149	0.0352	0.3903
GYG2	-0.0159	0.0360	0.3920
MED14	0.0137	0.0387	0.4019
ARMCX5	0.0137	0.0397	0.4054
HSFX1	-0.0144	0.0400	0.4063
RBM3	0.0139	0.0422	0.4132

© 2021 Davis EJ et al. *JAMA Neurology.*

GPRASP2	0.0131	0.0423	0.4132
RAB9A	-0.0141	0.0425	0.4141
HPRT1	0.0144	0.0430	0.4157
SYAP1	0.0134	0.0451	0.4197
SLC9A7	0.0128	0.0462	0.4233
DDX26B	0.0135	0.0466	0.4247
PHF8	-0.0136	0.0466	0.4247
PORCN	0.0136	0.0473	0.4257
PDHA1	0.0123	0.0478	0.4275
ARSD	0.0138	0.0510	0.4359
TCEAL5	0.0143	0.0512	0.4360
TAB3	0.0127	0.0546	0.4423
RBMX	0.0127	0.0555	0.4449
APLN	-0.0141	0.0575	0.4498
RPL10	0.0133	0.0583	0.4520
TIMM8A	0.0121	0.0594	0.4545
ZMYM3	-0.0136	0.0611	0.4579
TMEM187	-0.0132	0.0626	0.4612
PGRMC1	0.0125	0.0635	0.4639
NDUFA1	0.0128	0.0644	0.4646
COX7B	0.0122	0.0660	0.4684
IL3RA	-0.0125	0.0662	0.4684
GPC4	-0.0136	0.0664	0.4687
POLA1	-0.0137	0.0668	0.4703
CDKL5	0.0121	0.0673	0.4708
USP11	0.0125	0.0696	0.4761
GPRASP1	0.0124	0.0711	0.4795
RP1-177G6.2	-0.0131	0.0723	0.4824
TMLHE	0.0121	0.0738	0.4855
DYNLT3	0.0126	0.0740	0.4855
PHKA2	-0.0117	0.0742	0.4858
BCOR	-0.0127	0.0746	0.4865
BEX1	0.0125	0.0746	0.4865
MAP7D2	0.0124	0.0766	0.4904
TMSB4X	0.0131	0.0778	0.4922
STAG2	-0.0113	0.0795	0.4957
SPIN2B	0.0128	0.0800	0.4963
BEX5	0.0128	0.0809	0.4982
EMD	0.0124	0.0828	0.5018
SMC1A	-0.0141	0.0827	0.5018
RNF128	0.0122	0.0835	0.5030
BEX2	0.0119	0.0839	0.5046

© 2021 Davis EJ et al. *JAMA Neurology.*

ACRC	-0.0126	0.0848	0.5057
RAP2C	0.0111	0.0871	0.5099
SLC10A3	0.0117	0.0911	0.5181
CXorf26	0.0112	0.0914	0.5193
ABCB7	-0.0122	0.0926	0.5207
RRAGB	0.0118	0.0948	0.5242
ZNF81	-0.0111	0.0966	0.5285
UPRT	0.0111	0.0977	0.5306
ZNF711	0.0113	0.0975	0.5306
SPIN2A	-0.0112	0.1002	0.5335
EIF1AX	0.0112	0.1051	0.5406
KLF8	0.0118	0.1055	0.5411
FAM199X	0.0115	0.1095	0.5459
SLC38A5	0.0119	0.1120	0.5495
CXorf24	-0.0099	0.1170	0.5582
RENBP	-0.0106	0.1190	0.5618
ZBTB33	-0.0108	0.1215	0.5663
MBTPS2	0.0103	0.1230	0.5683
TCEANC	-0.0101	0.1237	0.5684
C1GALT1C1	-0.0114	0.1235	0.5684
ZNF41	0.0098	0.1294	0.5789
TFE3	-0.0110	0.1338	0.5850
CSAG1	-0.0100	0.1429	0.5955
GRIA3	0.0098	0.1459	0.5985
F8	0.0095	0.1475	0.6002
IRAK1	-0.0100	0.1477	0.6007
L1CAM	-0.0103	0.1480	0.6012
SCML2	-0.0106	0.1486	0.6018
PNMA3	0.0100	0.1508	0.6047
UBE2A	0.0099	0.1513	0.6051
USP9X	0.0106	0.1534	0.6071
XK	0.0096	0.1537	0.6073
PLXNB3	-0.0097	0.1541	0.6073
CD99L2	0.0092	0.1544	0.6073
FAM156A	0.0097	0.1557	0.6093
PNPLA4	0.0103	0.1560	0.6099
TSPAN7	0.0097	0.1575	0.6118
CXorf23	0.0097	0.1579	0.6118
BEX4	0.0099	0.1597	0.6141
RAB33A	-0.0091	0.1619	0.6155
GDI1	0.0102	0.1648	0.6193
IL2RG	-0.0105	0.1657	0.6199

© 2021 Davis EJ et al. *JAMA Neurology.*

ZBED1	-0.0098	0.1696	0.6228
VBP1	0.0091	0.1702	0.6241
SYTL4	-0.0096	0.1719	0.6264
PHF16	-0.0096	0.1728	0.6273
FAM50A	-0.0096	0.1745	0.6296
SLC35A2	0.0090	0.1764	0.6308
MAGED1	0.0092	0.1813	0.6360
TRAPPC2	-0.0096	0.1817	0.6361
TCEAL6	0.0091	0.1834	0.6380
TMEM185A	-0.0090	0.1842	0.6382
CUL4B	0.0095	0.1852	0.6389
STS	0.0087	0.1892	0.6422
GNL3L	0.0087	0.1921	0.6451
FAM155B	-0.0088	0.1934	0.6461
FUNDC1	0.0093	0.1935	0.6463
FGF13	0.0093	0.1970	0.6496
PLXNA3	-0.0084	0.1976	0.6503
VAMP7	0.0095	0.1991	0.6519
OTUD5	0.0083	0.1995	0.6520
TSPAN6	-0.0088	0.2024	0.6525
CCDC120	-0.0093	0.2037	0.6537
NAP1L2	0.0088	0.2098	0.6604
TBL1X	-0.0090	0.2110	0.6618
ARMCX1	0.0088	0.2110	0.6619
GSPT2	0.0080	0.2119	0.6625
RGAG4	0.0083	0.2129	0.6636
GPKOW	0.0100	0.2134	0.6639
GDPD2	-0.0090	0.2135	0.6639
EFNB1	-0.0081	0.2150	0.6649
ARSE	-0.0085	0.2159	0.6661
SH3KBP1	0.0076	0.2297	0.6782
GABRE	-0.0084	0.2301	0.6783
MID1	-0.0087	0.2326	0.6802
DLG3	0.0079	0.2344	0.6815
SAT1	-0.0081	0.2352	0.6821
PCDH19	0.0089	0.2367	0.6841
DUSP9	-0.0086	0.2398	0.6872
GK	0.0086	0.2413	0.6890
VSIG4	-0.0087	0.2447	0.6919
NDP	-0.0082	0.2459	0.6928
G6PD	0.0080	0.2483	0.6947
CHRDL1	0.0082	0.2505	0.6960

© 2021 Davis EJ et al. *JAMA Neurology.*

MAGIX	0.0080	0.2518	0.6978
KDM6A	-0.0086	0.2559	0.7011
CCDC160	-0.0083	0.2573	0.7025
HEPH	-0.0079	0.2578	0.7029
AL158821.1	-0.0075	0.2593	0.7037
LONRF3	-0.0076	0.2600	0.7043
SMS	0.0080	0.2660	0.7108
ATRX	-0.0082	0.2709	0.7143
HNRNPH2	0.0077	0.2709	0.7143
TCEAL7	0.0074	0.2720	0.7155
PDZD4	-0.0076	0.2727	0.7163
EFHC2	-0.0078	0.2734	0.7164
TCEAL1	-0.0074	0.2740	0.7164
FAM127B	0.0072	0.2748	0.7168
SH3BGRL	0.0081	0.2748	0.7168
KDM5C	-0.0080	0.2775	0.7179
FIGF	-0.0072	0.2838	0.7231
SUV39H1	0.0072	0.2857	0.7250
ARMCX3	0.0073	0.2883	0.7268
CXorf38	0.0077	0.2918	0.7285
PNMA6C	0.0063	0.2961	0.7313
KCNE1L	-0.0064	0.2970	0.7319
HMGN5	-0.0073	0.2978	0.7328
IDS	0.0070	0.3001	0.7344
ZRSR2	-0.0071	0.3025	0.7364
AIFM1	0.0071	0.3086	0.7386
GPR34	0.0073	0.3078	0.7386
CHM	-0.0066	0.3091	0.7387
CETN2	0.0073	0.3105	0.7399
PRAF2	0.0072	0.3122	0.7414
ZCCHC12	0.0070	0.3130	0.7422
ARHGEF6	-0.0074	0.3145	0.7427
BGN	-0.0068	0.3192	0.7465
SLC25A5	0.0070	0.3204	0.7474
PHKA1	-0.0068	0.3223	0.7487
ZDHHC15	0.0062	0.3232	0.7494
ARMCX2	0.0067	0.3239	0.7498
WDR44	0.0070	0.3243	0.7499
PABPC1L2A	-0.0066	0.3274	0.7522
SSR4	0.0069	0.3284	0.7527
NGFRAP1	0.0067	0.3299	0.7534
PRPS2	0.0065	0.3305	0.7537

© 2021 Davis EJ et al. *JAMA Neurology.*

PPP2R3B	-0.0068	0.3320	0.7552
IGSF1	-0.0065	0.3345	0.7569
UPF3B	-0.0072	0.3407	0.7604
TBC1D8B	-0.0063	0.3412	0.7608
DRP2	0.0066	0.3433	0.7614
ARMCX4	0.0064	0.3562	0.7693
UXT	-0.0062	0.3646	0.7751
ATP6AP2	0.0063	0.3669	0.7761
CXorf40B	0.0062	0.3721	0.7780
SRPX	0.0062	0.3699	0.7780
NONO	-0.0066	0.3746	0.7780
UTP14A	0.0057	0.3727	0.7780
NAP1L3	0.0061	0.3768	0.7789
DMRTC1B	-0.0061	0.3766	0.7789
RAI2	-0.0060	0.3789	0.7805
NUDT11	0.0061	0.3843	0.7835
ARX	-0.0060	0.3849	0.7835
ZFX	-0.0061	0.3852	0.7838
MCTS1	0.0061	0.3858	0.7844
ALG13	0.0059	0.3892	0.7867
SMPX	0.0060	0.3903	0.7871
HDHD1	0.0055	0.3947	0.7892
ARHGEF9	0.0057	0.3979	0.7909
TCEAL8	-0.0058	0.3993	0.7915
EBP	0.0060	0.4001	0.7921
ZNF449	0.0057	0.4007	0.7923
CCDC22	0.0054	0.4009	0.7924
RBM10	-0.0054	0.4033	0.7939
ARHGAP4	0.0057	0.4050	0.7946
PNCK	0.0058	0.4086	0.7971
FHL1	0.0061	0.4105	0.7983
LRCH2	-0.0055	0.4135	0.7995
MAP7D3	-0.0055	0.4140	0.7996
AFF2	-0.0053	0.4163	0.8008
OPHN1	-0.0059	0.4162	0.8008
SLC6A8	-0.0055	0.4177	0.8015
CXorf40A	0.0052	0.4190	0.8018
SNX12	0.0055	0.4190	0.8018
MAGEH1	0.0056	0.4195	0.8018
FMR1	0.0057	0.4197	0.8018
FAM122B	0.0054	0.4219	0.8032
HUWE1	-0.0056	0.4227	0.8038

© 2021 Davis EJ et al. *JAMA Neurology.*

NHS	-0.0057	0.4229	0.8039
PCSK1N	0.0061	0.4250	0.8051
ZNF275	0.0056	0.4259	0.8056
MAGED2	0.0056	0.4265	0.8058
FAM70A	-0.0054	0.4285	0.8064
SHROOM2	0.0050	0.4377	0.8121
TAF1	0.0059	0.4410	0.8133
TMEM47	-0.0052	0.4416	0.8133
SPIN3	0.0053	0.4422	0.8138
FAM3A	0.0054	0.4426	0.8138
IL13RA1	0.0052	0.4526	0.8198
MAOA	0.0053	0.4585	0.8224
APOO	0.0053	0.4588	0.8226
TCEAL2	0.0051	0.4599	0.8230
RBMX2	-0.0051	0.4615	0.8234
GPR143	0.0052	0.4631	0.8240
MUM1L1	-0.0050	0.4629	0.8240
RPL39	-0.0051	0.4664	0.8258
IKBKG	0.0050	0.4712	0.8278
FAM120C	-0.0053	0.4720	0.8282
LAS1L	0.0050	0.4745	0.8289
CITED1	0.0052	0.4745	0.8290
SMARCA1	-0.0051	0.4760	0.8297
IDH3G	0.0050	0.4790	0.8307
USP51	0.0050	0.4852	0.8332
NLGN3	0.0050	0.4873	0.8345
PRPS1	0.0048	0.4911	0.8363
HDAC6	-0.0050	0.4927	0.8370
ATP2B3	0.0045	0.5058	0.8443
APEX2	0.0045	0.5068	0.8446
RIBC1	-0.0047	0.5072	0.8449
PJA1	0.0046	0.5080	0.8453
PRRG1	-0.0045	0.5126	0.8474
TXLNG	0.0042	0.5126	0.8474
MAGED4	-0.0043	0.5159	0.8494
SLC25A6	0.0043	0.5221	0.8524
CXorf57	-0.0044	0.5271	0.8549
PIM2	0.0043	0.5274	0.8551
PQBP1	0.0042	0.5277	0.8552
PSMD10	0.0048	0.5290	0.8560
ZNF185	0.0043	0.5325	0.8579
WWC3	-0.0039	0.5346	0.8584

© 2021 Davis EJ et al. *JAMA Neurology.*

CXorf36	-0.0043	0.5348	0.8585
RPS6KA3	-0.0041	0.5445	0.8641
TMEM164	-0.0042	0.5449	0.8644
RP11-274K13.2	0.0040	0.5499	0.8672
CA5B	0.0041	0.5519	0.8684
GPM6B	-0.0041	0.5521	0.8685
HS6ST2	0.0040	0.5542	0.8692
ZNF674	0.0040	0.5560	0.8702
SLC25A53	0.0040	0.5584	0.8713
PRDX4	-0.0044	0.5587	0.8714
GTPBP6	-0.0041	0.5593	0.8719
GJB1	0.0038	0.5623	0.8733
LAMP2	-0.0039	0.5683	0.8760
CSF2RA	0.0037	0.5698	0.8767
GRIPAP1	0.0038	0.5718	0.8777
HSD17B10	0.0040	0.5733	0.8784
PHF6	-0.0040	0.5741	0.8785
PNMA6A	-0.0039	0.5741	0.8785
ACOT9	0.0039	0.5745	0.8785
WDR45	-0.0040	0.5750	0.8786
MMGT1	0.0039	0.5810	0.8809
CXorf69	-0.0040	0.5831	0.8819
FAM123B	-0.0040	0.5835	0.8821
CTPS2	-0.0040	0.5853	0.8827
SCML1	0.0036	0.5895	0.8844
DHRSX	0.0038	0.5931	0.8856
SYP	0.0037	0.5951	0.8862
MCF2	-0.0037	0.5951	0.8862
TCEAL4	0.0036	0.5954	0.8863
CFP	0.0035	0.5966	0.8865
PRRG3	0.0035	0.6019	0.8884
ODZ1	0.0038	0.6031	0.8891
PIGA	-0.0036	0.6054	0.8896
AMMECR1	-0.0035	0.6063	0.8896
RAB39B	0.0035	0.6064	0.8897
MAGEE1	0.0036	0.6065	0.8897
EIF2S3	-0.0034	0.6073	0.8900
SASH3	-0.0038	0.6113	0.8919
KLHL4	0.0035	0.6115	0.8919
AP1S2	0.0036	0.6119	0.8921
CD99	-0.0035	0.6121	0.8922
BRWD3	-0.0035	0.6148	0.8936

© 2021 Davis EJ et al. *JAMA Neurology.*

XIAP	-0.0034	0.6166	0.8941
ACSL4	0.0033	0.6206	0.8955
ZDHHC9	0.0036	0.6206	0.8955
LDOC1	0.0033	0.6223	0.8962
ZXDB	0.0034	0.6228	0.8964
SLC25A14	0.0034	0.6261	0.8974
RAB9B	0.0032	0.6271	0.8975
GABRA3	-0.0033	0.6278	0.8979
FAM156B	0.0034	0.6315	0.8999
WAS	0.0033	0.6360	0.9014
BCORL1	-0.0035	0.6363	0.9015
FAM122C	0.0034	0.6383	0.9024
ATG4A	-0.0032	0.6406	0.9031
TIMM17B	0.0035	0.6419	0.9035
RPL36A	-0.0031	0.6444	0.9050
KLHL15	-0.0031	0.6462	0.9054
MTM1	-0.0033	0.6512	0.9071
GPR173	0.0032	0.6548	0.9082
MORF4L2	0.0031	0.6589	0.9092
FRMPD4	0.0030	0.6602	0.9097
MAGEE2	-0.0029	0.6624	0.9107
ZNF75D	0.0029	0.6637	0.9112
MAGT1	0.0029	0.6640	0.9112
ABCD1	-0.0027	0.6851	0.9193
FATE1	-0.0026	0.6887	0.9205
MOSPD1	-0.0028	0.6904	0.9210
IL1RAPL1	-0.0029	0.6993	0.9245
LAGE3	0.0026	0.7021	0.9255
SLC9A6	-0.0025	0.7044	0.9266
CHIC1	-0.0026	0.7053	0.9269
MTCP1	0.0026	0.7072	0.9277
RPS4X	0.0026	0.7109	0.9289
OFD1	-0.0025	0.7123	0.9294
SLITRK4	-0.0025	0.7134	0.9297
TIMP1	-0.0025	0.7152	0.9299
MAMLD1	0.0025	0.7216	0.9320
FLNA	0.0023	0.7240	0.9330
FAAH2	-0.0025	0.7271	0.9341
RPGR	-0.0025	0.7281	0.9344
COL4A5	0.0024	0.7285	0.9344
ARMCX6	0.0024	0.7300	0.9346
F8A1	-0.0021	0.7352	0.9362

© 2021 Davis EJ et al. *JAMA Neurology.*

TCEAL3	-0.0023	0.7356	0.9363
SEPT	-0.0023	0.7418	0.9385
MTCP1NB	-0.0024	0.7435	0.9390
GEMIN8	-0.0023	0.7466	0.9393
ENOX2	0.0022	0.7513	0.9409
BTK	0.0023	0.7514	0.9410
FUNDC2	-0.0022	0.7569	0.9422
RGN	-0.0021	0.7575	0.9425
DIAPH2	0.0020	0.7586	0.9427
FGD1	-0.0021	0.7611	0.9434
SLC16A2	0.0022	0.7622	0.9436
PIR	0.0020	0.7626	0.9437
ZNF182	-0.0020	0.7632	0.9439
GLA	-0.0020	0.7660	0.9451
UBL4A	0.0022	0.7667	0.9452
CNKSR2	-0.0020	0.7686	0.9454
PDK3	-0.0019	0.7769	0.9479
ZMAT1	0.0019	0.7782	0.9480
MTMR1	-0.0021	0.7790	0.9482
OCRL	0.0018	0.7826	0.9488
SLITRK2	-0.0020	0.7833	0.9488
KCND1	-0.0017	0.7867	0.9494
ATP6AP1	0.0018	0.7883	0.9499
PLP2	-0.0018	0.7908	0.9505
APOOL	-0.0018	0.7935	0.9518
CHST7	0.0017	0.7992	0.9537
SLC25A43	0.0016	0.7992	0.9537
DDX3X	-0.0019	0.8023	0.9544
ARSF	-0.0017	0.8058	0.9555
KAL1	0.0017	0.8131	0.9577
BCAP31	-0.0016	0.8146	0.9578
HTATSF1	-0.0016	0.8194	0.9585
NKAP	-0.0017	0.8226	0.9592
PLCXD1	-0.0016	0.8269	0.9603
NLGN4X	0.0014	0.8342	0.9624
RBM41	0.0014	0.8415	0.9642
MSL3	-0.0014	0.8423	0.9645
NUDT10	0.0014	0.8464	0.9657
IGBP1	0.0013	0.8467	0.9659
RHOXF1	-0.0012	0.8479	0.9663
FAM127C	-0.0012	0.8504	0.9667
DACH2	0.0014	0.8505	0.9667

© 2021 Davis EJ et al. *JAMA Neurology.*

DMRTC1	0.0012	0.8579	0.9687
SHROOM4	0.0011	0.8616	0.9702
FAM133A	0.0013	0.8619	0.9702
AMOT	-0.0012	0.8674	0.9717
BHLHB9	0.0011	0.8677	0.9717
PCDH11X	0.0010	0.8761	0.9740
UBA1	-0.0011	0.8778	0.9745
RBBP7	-0.0010	0.8790	0.9748
PPP1R3F	-0.0011	0.8826	0.9754
AKAP17A	-0.0010	0.8826	0.9754
MPP1	-0.0010	0.8824	0.9754
HAUS7	0.0010	0.8876	0.9765
RLIM	-0.0009	0.8900	0.9768
MID2	0.0010	0.8926	0.9777
TMSB15B	-0.0009	0.8952	0.9783
WDR13	-0.0010	0.8970	0.9791
NXT2	-0.0008	0.9033	0.9804
MOSPD2	0.0008	0.9066	0.9816
CASK	-0.0008	0.9089	0.9817
REPS2	0.0008	0.9100	0.9819
STARD8	-0.0007	0.9148	0.9831
KIAA2022	0.0007	0.9148	0.9831
YIPF6	0.0007	0.9182	0.9840
PAK3	-0.0008	0.9185	0.9842
ERAS	-0.0007	0.9207	0.9847
PLP1	0.0007	0.9218	0.9850
PGK1	0.0007	0.9230	0.9850
MAOB	-0.0007	0.9237	0.9851
ZC3H12B	-0.0006	0.9276	0.9863
INE1	-0.0006	0.9284	0.9865
UBQLN2	0.0006	0.9291	0.9868
FAM58A	0.0006	0.9311	0.9872
ZNF673	-0.0006	0.9312	0.9873
PABPC1L2B	0.0006	0.9317	0.9874
TRMT2B	0.0005	0.9319	0.9875
TMEM35	-0.0006	0.9320	0.9875
F8A2	-0.0005	0.9378	0.9883
CYBB	0.0006	0.9389	0.9886
CLCN4	0.0005	0.9412	0.9891
RNF113A	0.0005	0.9426	0.9892
TSPYL2	-0.0005	0.9454	0.9895
TBC1D25	0.0004	0.9474	0.9901

© 2021 Davis EJ et al. *JAMA Neurology.*

HDAC8	-0.0004	0.9543	0.9920
CXorf1	0.0003	0.9572	0.9923
ZC4H2	-0.0003	0.9580	0.9926
TRO	-0.0004	0.9586	0.9926
KLHL13	-0.0004	0.9601	0.9927
MSN	0.0003	0.9624	0.9930
ZNF630	-0.0003	0.9632	0.9932
NSDHL	0.0003	0.9644	0.9935
SYN1	-0.0003	0.9642	0.9935
NDUFB11	0.0003	0.9647	0.9935
MAGEC3	-0.0003	0.9665	0.9938
SRPK3	0.0003	0.9678	0.9943
CYSLTR1	-0.0002	0.9749	0.9954
DCX	-0.0002	0.9730	0.9954
IQSEC2	-0.0002	0.9733	0.9954
OGT	0.0002	0.9732	0.9954
CDK16	0.0002	0.9768	0.9959
NAA10	-0.0002	0.9813	0.9974
FAM104B	-0.0001	0.9838	0.9975
THOC2	0.0002	0.9834	0.9975
NKRF	0.0001	0.9878	0.9984
DKC1	0.0001	0.9886	0.9986
PCYT1B	-0.0001	0.9920	0.9992
ARAF	-0.0001	0.9937	0.9993
HCCS	0.0000	0.9944	0.9995
F8A3	0.0000	0.9970	0.9998
PIN4	0.0000	0.9976	0.9998
ATP11C	0.0000	0.9994	0.9999

© 2021 Davis EJ et al. *JAMA Neurology.*

eTable 4. Global cognitive decline and NFT in men compared to women, adjusted by age and education		
**Measure**	**Beta**	**Pvalue**
Global cognitive decline (women)	-0.180	0.86
NFT (women)	-0.056	0.07

© 2021 Davis EJ et al. *JAMA Neurology.*

**eTable 5. Association of X chromosome gene expression with neurofibrillary tangles in women, adjusted by age and education.** FDR-adjusted p-values for genome-wide correction shown.			
**Gene**	**Beta**	**Pvalue**	**Pvalue Adjusted**
ELK1	0.0748	0.0001	0.1176
PDZD11	-0.0675	0.0004	0.2450
SMS	-0.0654	0.0005	0.2555
PPEF1	-0.0610	0.0010	0.2753
LONRF3	0.0596	0.0015	0.3155
FOXO4	0.0591	0.0019	0.3199
MCTS1	-0.0557	0.0026	0.3364
GPRASP1	-0.0555	0.0029	0.3430
MAGED4B	0.0561	0.0028	0.3430
MAGEH1	-0.0541	0.0042	0.3665
FGF13	-0.0535	0.0042	0.3665
RRAGB	-0.0530	0.0046	0.3723
CSTF2	-0.0536	0.0049	0.3730
ATRX	0.0505	0.0054	0.3766
TMLHE	-0.0522	0.0056	0.3787
ZBED1	0.0514	0.0058	0.3806
DYNLT3	-0.0499	0.0067	0.3965
COX7B	-0.0515	0.0080	0.4038
OTUD5	-0.0497	0.0111	0.4108
G6PD	-0.0487	0.0102	0.4108
PDHA1	-0.0502	0.0117	0.4144
PRPS1	-0.0472	0.0123	0.4190
NAA10	-0.0478	0.0123	0.4190
ARSE	0.0462	0.0134	0.4228
HPRT1	-0.0457	0.0139	0.4262
MBTPS2	-0.0470	0.0147	0.4287
PIM2	-0.0467	0.0146	0.4287
TSPAN7	-0.0466	0.0169	0.4371
NHS	-0.0447	0.0184	0.4411
MOSPD1	0.0442	0.0185	0.4416
TXLNG	-0.0461	0.0195	0.4485
FAM127A	-0.0454	0.0204	0.4528
THOC2	0.0426	0.0212	0.4558
GRIPAP1	-0.0441	0.0213	0.4558
PLXNB3	0.0434	0.0217	0.4580
EIF2S3	-0.0438	0.0218	0.4580
VBP1	-0.0445	0.0231	0.4619
KCNE1L	0.0469	0.0238	0.4646
MED12	0.0436	0.0239	0.4652
TSC22D3	0.0423	0.0246	0.4663

© 2021 Davis EJ et al. *JAMA Neurology.*

PCYT1B	0.0422	0.0250	0.4677
ZNF673	0.0433	0.0263	0.4737
SRPK3	0.0411	0.0282	0.4802
CSF2RA	0.0427	0.0306	0.4806
SPIN2B	-0.0401	0.0291	0.4806
HSFX1	0.0400	0.0310	0.4806
CXorf1	0.0419	0.0299	0.4806
KAL1	-0.0402	0.0336	0.4806
ATP6AP2	-0.0403	0.0307	0.4806
TMSB4X	-0.0392	0.0301	0.4806
PQBP1	0.0417	0.0342	0.4822
CCDC120	0.0389	0.0343	0.4825
SCML2	0.0382	0.0356	0.4851
PRAF2	-0.0394	0.0358	0.4855
NGFRAP1	-0.0389	0.0373	0.4885
UXT	0.0401	0.0381	0.4885
GPRASP2	-0.0406	0.0376	0.4885
HCFC1	0.0383	0.0384	0.4885
TSPAN6	0.0395	0.0383	0.4885
ABCD1	0.0402	0.0402	0.4934
BEX4	-0.0387	0.0406	0.4938
TAZ	0.0393	0.0412	0.4950
ZCCHC12	-0.0377	0.0472	0.5114
BEX2	-0.0374	0.0473	0.5116
GABRE	-0.0365	0.0511	0.5207
PDK3	-0.0370	0.0553	0.5318
PGK1	-0.0348	0.0577	0.5349
TSR2	-0.0355	0.0598	0.5407
ZDHHC9	0.0348	0.0600	0.5408
HDHD1	0.0369	0.0606	0.5420
VMA21	-0.0349	0.0619	0.5431
SYTL4	0.0341	0.0652	0.5491
AKAP17A	0.0352	0.0656	0.5501
TIMM8A	-0.0367	0.0675	0.5530
RAB9A	0.0344	0.0687	0.5552
CCDC160	0.0335	0.0693	0.5564
TRAPPC2	0.0341	0.0700	0.5574
TBC1D8B	0.0351	0.0709	0.5587
RP1-177G6.2	0.0335	0.0727	0.5602
CXorf38	-0.0324	0.0739	0.5627
ACSL4	-0.0343	0.0766	0.5696
PHF16	0.0343	0.0766	0.5696

© 2021 Davis EJ et al. *JAMA Neurology.*

MID2	-0.0326	0.0775	0.5705
GK	-0.0324	0.0771	0.5705
TAF9B	-0.0328	0.0781	0.5718
RLIM	-0.0335	0.0783	0.5726
IL1RAPL1	0.0318	0.0791	0.5736
SRPX	0.0334	0.0808	0.5739
EMD	-0.0325	0.0822	0.5748
SHROOM2	-0.0342	0.0830	0.5757
GNL3L	-0.0328	0.0857	0.5796
FAM156B	-0.0322	0.0865	0.5801
HS6ST2	-0.0332	0.0872	0.5811
SLC9A7	-0.0343	0.0878	0.5824
HMGN5	0.0322	0.0884	0.5844
RNF128	-0.0316	0.0894	0.5868
AL158821.1	0.0328	0.0913	0.5891
ASMTL	-0.0314	0.0954	0.5947
PLP1	0.0314	0.0978	0.5991
LAGE3	-0.0309	0.0985	0.6005
GPR173	0.0305	0.1021	0.6019
CXorf24	0.0323	0.1025	0.6029
F8	-0.0316	0.1031	0.6044
RP11-274K13.2	-0.0315	0.1035	0.6046
NDUFB11	-0.0309	0.1035	0.6046
CITED1	-0.0295	0.1055	0.6080
RBMX	-0.0313	0.1057	0.6081
ZNF674	-0.0306	0.1059	0.6086
SSR4	-0.0317	0.1122	0.6166
RPL10	-0.0301	0.1140	0.6196
PRKX	0.0310	0.1150	0.6208
ATP6AP1	-0.0306	0.1152	0.6208
MTCP1NB	0.0294	0.1156	0.6213
KDM6A	0.0279	0.1180	0.6245
SPIN3	-0.0299	0.1217	0.6278
MSL3	0.0294	0.1226	0.6286
CLIC2	-0.0287	0.1231	0.6292
SLC6A8	0.0292	0.1230	0.6292
SMC1A	0.0275	0.1230	0.6292
PSMD10	-0.0281	0.1229	0.6292
CXorf69	0.0283	0.1245	0.6309
RAP2C	-0.0300	0.1248	0.6316
ARSF	0.0283	0.1257	0.6327
ZFX	0.0293	0.1260	0.6328

© 2021 Davis EJ et al. *JAMA Neurology.*

ZXDB	-0.0290	0.1271	0.6346
F8A2	0.0297	0.1304	0.6368
NAP1L2	-0.0283	0.1304	0.6368
CXorf56	-0.0290	0.1318	0.6377
F8A3	0.0296	0.1333	0.6401
DNASE1L1	-0.0296	0.1359	0.6425
SYP	-0.0281	0.1362	0.6425
ZMYM3	0.0279	0.1365	0.6430
CXorf36	0.0280	0.1365	0.6430
PORCN	-0.0282	0.1435	0.6530
NAP1L3	-0.0275	0.1475	0.6552
CNKSR2	0.0281	0.1470	0.6552
GPC4	0.0262	0.1484	0.6571
MED14	-0.0271	0.1557	0.6664
GJB1	0.0276	0.1613	0.6710
ARMCX2	-0.0267	0.1619	0.6714
CXorf23	0.0263	0.1632	0.6723
GLA	0.0263	0.1653	0.6741
RENBP	0.0264	0.1659	0.6741
RBM3	-0.0261	0.1673	0.6755
POLA1	0.0251	0.1692	0.6776
RBBP7	0.0273	0.1693	0.6776
WWC3	0.0273	0.1716	0.6797
SCML1	-0.0258	0.1730	0.6813
YIPF6	0.0253	0.1750	0.6835
TCEAL5	-0.0249	0.1757	0.6846
RPGR	0.0258	0.1770	0.6859
ENOX2	0.0260	0.1802	0.6895
BRWD3	0.0257	0.1815	0.6909
CASK	-0.0253	0.1855	0.6946
MMGT1	-0.0252	0.1888	0.6962
PHF8	0.0247	0.1912	0.6975
CXorf26	0.0252	0.1927	0.6985
UPF3B	0.0236	0.1935	0.6995
USP9X	-0.0238	0.1956	0.7020
APOOL	-0.0247	0.1985	0.7043
ABCB7	-0.0233	0.2015	0.7063
FAM155B	0.0248	0.2027	0.7072
AP1S2	-0.0237	0.2029	0.7073
GEMIN8	-0.0238	0.2054	0.7094
KLF8	-0.0233	0.2059	0.7099
VAMP7	-0.0232	0.2092	0.7128

© 2021 Davis EJ et al. *JAMA Neurology.*

SLC25A5	-0.0235	0.2123	0.7152
XIAP	-0.0232	0.2181	0.7201
GDI1	-0.0225	0.2188	0.7205
PLS3	-0.0232	0.2187	0.7205
SLC25A14	-0.0231	0.2205	0.7222
HNRNPH2	-0.0229	0.2233	0.7241
CHRDL1	-0.0228	0.2230	0.7241
ZC3H12B	0.0232	0.2232	0.7241
FAM127C	0.0241	0.2258	0.7260
FUNDC2	0.0228	0.2260	0.7261
PLCXD1	0.0218	0.2299	0.7293
FATE1	0.0241	0.2301	0.7296
PLP2	0.0224	0.2358	0.7336
ZBTB33	-0.0222	0.2369	0.7336
DLG3	-0.0225	0.2365	0.7336
NKRF	-0.0224	0.2379	0.7336
APOO	-0.0217	0.2411	0.7353
FRMPD3	-0.0225	0.2411	0.7353
PCDH11X	-0.0227	0.2414	0.7354
NXT2	0.0221	0.2426	0.7363
APLN	0.0211	0.2465	0.7373
IRAK1	0.0221	0.2457	0.7373
FGD1	0.0222	0.2475	0.7378
PNPLA4	-0.0215	0.2482	0.7383
USP11	-0.0216	0.2528	0.7414
MAGEE1	-0.0214	0.2549	0.7427
PPP2R3B	0.0212	0.2558	0.7437
MORF4L2	-0.0215	0.2559	0.7438
SLC25A43	-0.0227	0.2568	0.7440
SH3KBP1	-0.0228	0.2571	0.7440
MAGED2	-0.0213	0.2579	0.7440
MORC4	0.0215	0.2590	0.7447
ARMCX6	0.0213	0.2620	0.7467
PRRG1	0.0215	0.2624	0.7471
FAM3A	-0.0208	0.2648	0.7492
ALG13	-0.0212	0.2654	0.7498
UBL4A	0.0206	0.2659	0.7499
ATP2B3	-0.0215	0.2668	0.7504
UBE2A	-0.0211	0.2693	0.7518
FAM156A	-0.0212	0.2692	0.7518
MID1IP1	0.0215	0.2698	0.7522
SLC25A6	-0.0213	0.2737	0.7552

© 2021 Davis EJ et al. *JAMA Neurology.*

WDR45	0.0204	0.2739	0.7554
RPS6KA3	-0.0212	0.2755	0.7555
OPHN1	-0.0199	0.2779	0.7563
UTP14A	0.0214	0.2787	0.7568
IDS	-0.0208	0.2795	0.7570
BEX1	-0.0205	0.2795	0.7570
ARMCX4	-0.0207	0.2804	0.7574
DMRTC1B	0.0202	0.2822	0.7599
STARD8	0.0201	0.2864	0.7621
GPKOW	-0.0191	0.2865	0.7621
SLC16A2	-0.0199	0.2869	0.7625
ARHGEF9	-0.0205	0.2880	0.7631
COL4A5	0.0200	0.2884	0.7635
MECP2	0.0206	0.2888	0.7637
PCDH19	0.0193	0.2909	0.7651
TCEAL7	-0.0203	0.2925	0.7666
SEPT	-0.0200	0.2979	0.7689
MAGT1	-0.0200	0.2991	0.7697
TIMM17B	-0.0190	0.2992	0.7697
PNMA3	-0.0193	0.3037	0.7711
ARX	-0.0200	0.3036	0.7711
ARMCX5	-0.0196	0.3042	0.7714
FIGF	0.0198	0.3051	0.7714
EIF1AX	-0.0194	0.3077	0.7723
RGN	-0.0194	0.3088	0.7731
KLHL13	0.0191	0.3111	0.7752
INE1	-0.0188	0.3135	0.7767
IKBKG	-0.0191	0.3146	0.7777
PNMA6C	0.0211	0.3161	0.7784
PRRG3	-0.0200	0.3173	0.7791
MAP7D2	-0.0188	0.3187	0.7807
SLC35A2	-0.0191	0.3202	0.7814
LAMP2	0.0188	0.3220	0.7823
AMMECR1	0.0186	0.3252	0.7840
NONO	0.0175	0.3318	0.7878
FAM50A	0.0183	0.3374	0.7913
RBM41	0.0179	0.3427	0.7945
SLC38A5	0.0174	0.3439	0.7951
WDR13	-0.0172	0.3464	0.7955
GPM6B	0.0177	0.3470	0.7960
GDPD2	0.0174	0.3480	0.7964
ZNF81	-0.0178	0.3506	0.7982

© 2021 Davis EJ et al. *JAMA Neurology.*

C1GALT1C1	0.0171	0.3510	0.7984
DRP2	-0.0174	0.3640	0.8039
KLHL15	-0.0173	0.3662	0.8048
FAM127B	0.0176	0.3687	0.8058
FAM199X	-0.0172	0.3697	0.8062
NSDHL	-0.0170	0.3712	0.8068
PIR	-0.0180	0.3736	0.8083
ZNF75D	-0.0174	0.3739	0.8083
BCORL1	0.0162	0.3775	0.8106
NKAP	0.0160	0.3777	0.8108
DIAPH2	-0.0175	0.3779	0.8109
HDAC8	0.0171	0.3790	0.8115
CXorf40B	-0.0168	0.3810	0.8120
RAB33A	0.0171	0.3806	0.8120
ARAF	0.0163	0.3855	0.8144
GRIA3	-0.0170	0.3869	0.8152
CYSLTR1	0.0162	0.3901	0.8174
TAF1	-0.0158	0.3903	0.8177
FAM122B	-0.0165	0.3908	0.8178
STAG2	0.0166	0.3979	0.8200
MAOA	-0.0159	0.3986	0.8205
ZNF182	0.0164	0.4001	0.8209
BTK	-0.0157	0.4004	0.8210
KLHL4	0.0160	0.4060	0.8242
ZNF630	-0.0160	0.4072	0.8246
TSPYL2	-0.0158	0.4147	0.8293
CHM	0.0156	0.4216	0.8323
AFF2	-0.0157	0.4290	0.8356
SLC25A53	-0.0151	0.4303	0.8363
MAOB	-0.0140	0.4352	0.8382
SH3BGRL	-0.0140	0.4391	0.8399
GPR143	-0.0147	0.4395	0.8400
AMOT	0.0144	0.4410	0.8403
NLGN3	0.0142	0.4445	0.8421
PGRMC1	-0.0150	0.4450	0.8421
CYBB	-0.0138	0.4466	0.8424
PNMA5	-0.0142	0.4533	0.8459
RPL36A	0.0145	0.4542	0.8468
PRPS2	-0.0143	0.4547	0.8474
IQSEC2	0.0140	0.4560	0.8480
KCND1	-0.0148	0.4593	0.8487
ATG4A	-0.0140	0.4637	0.8510

© 2021 Davis EJ et al. *JAMA Neurology.*

PDZD4	-0.0140	0.4642	0.8512
FAM104B	0.0137	0.4665	0.8525
OCRL	0.0141	0.4694	0.8534
ARSD	0.0136	0.4695	0.8534
MSN	-0.0131	0.4703	0.8538
FHL1	-0.0131	0.4714	0.8543
SLC10A3	0.0137	0.4731	0.8548
HDAC6	0.0132	0.4766	0.8559
GYG2	0.0128	0.4828	0.8586
MAGED1	-0.0133	0.4827	0.8586
BHLHB9	-0.0134	0.4844	0.8598
BEX5	-0.0127	0.4894	0.8621
SUV39H1	0.0130	0.4954	0.8643
PABPC1L2A	-0.0131	0.4971	0.8652
MPP1	-0.0130	0.4982	0.8659
SLITRK4	0.0132	0.4983	0.8660
FAM133A	0.0121	0.5012	0.8672
EBP	-0.0125	0.5015	0.8673
PIGA	-0.0123	0.5077	0.8698
PCSK1N	-0.0121	0.5110	0.8716
LRCH2	-0.0127	0.5132	0.8724
DDX3X	0.0119	0.5127	0.8724
CSAG1	0.0127	0.5141	0.8728
CD99	0.0121	0.5203	0.8752
ZNF449	-0.0123	0.5241	0.8768
HMGB3	-0.0121	0.5256	0.8768
FAM58A	0.0117	0.5271	0.8772
MAP7D3	0.0119	0.5299	0.8777
PTCHD1	0.0119	0.5343	0.8791
ERAS	-0.0117	0.5360	0.8797
MAGIX	-0.0114	0.5402	0.8815
FUNDC1	-0.0113	0.5440	0.8828
VSIG4	-0.0110	0.5449	0.8834
MID1	-0.0112	0.5450	0.8835
TCEAL6	-0.0111	0.5607	0.8880
SMPX	0.0109	0.5660	0.8895
DHRSX	0.0109	0.5652	0.8895
NDP	-0.0107	0.5677	0.8903
MAGEE2	-0.0109	0.5748	0.8920
SYN1	0.0108	0.5783	0.8932
PABPC1L2B	-0.0105	0.5858	0.8959
FAM70A	-0.0104	0.5864	0.8962

© 2021 Davis EJ et al. *JAMA Neurology.*

RBMX2	-0.0102	0.5877	0.8965
PHKA1	-0.0103	0.5906	0.8981
WBP5	0.0103	0.5911	0.8982
IL13RA1	0.0101	0.5921	0.8984
RAB9B	-0.0105	0.5925	0.8985
CUL4B	0.0100	0.5932	0.8987
TBL1X	0.0097	0.5965	0.8998
UBA1	0.0099	0.5990	0.9010
FAM120C	0.0096	0.5991	0.9010
TFE3	-0.0096	0.6031	0.9017
BRCC3	-0.0100	0.6039	0.9017
RHOXF1	-0.0109	0.6086	0.9035
PLXNA3	-0.0099	0.6108	0.9047
ARHGEF6	0.0092	0.6121	0.9051
PNCK	-0.0095	0.6171	0.9075
ATP11C	0.0092	0.6194	0.9083
TMSB15B	0.0093	0.6281	0.9110
OGT	-0.0088	0.6378	0.9134
RBM10	-0.0091	0.6388	0.9136
FAM122C	-0.0085	0.6399	0.9141
CXorf40A	-0.0091	0.6440	0.9150
RP11-706O15.1	-0.0085	0.6443	0.9152
IGSF1	0.0089	0.6444	0.9153
DUSP9	0.0085	0.6475	0.9158
ITM2A	0.0085	0.6510	0.9165
MOSPD2	0.0086	0.6584	0.9194
IL2RG	-0.0079	0.6600	0.9195
MTCP1	0.0081	0.6609	0.9199
PPP1R3F	-0.0081	0.6620	0.9203
MCF2	-0.0081	0.6657	0.9217
USP51	0.0079	0.6661	0.9217
BCOR	0.0079	0.6695	0.9236
ZMAT1	-0.0081	0.6717	0.9241
ZDHHC15	-0.0084	0.6719	0.9241
SLC9A6	0.0082	0.6760	0.9257
DACH2	-0.0077	0.6806	0.9266
PHF6	0.0077	0.6792	0.9266
CDKL5	-0.0079	0.6878	0.9285
ACRC	-0.0074	0.6890	0.9289
UPRT	-0.0075	0.6921	0.9299
TRO	-0.0072	0.6961	0.9313
TAB3	-0.0076	0.6959	0.9313

© 2021 Davis EJ et al. *JAMA Neurology.*

EFHC2	0.0073	0.6972	0.9313
XK	-0.0073	0.7017	0.9325
ACOT9	0.0074	0.7023	0.9325
AIFM1	0.0073	0.7015	0.9325
PHKA2	0.0074	0.7062	0.9335
MUM1L1	-0.0073	0.7052	0.9335
TCEANC	-0.0074	0.7074	0.9337
RAI2	0.0071	0.7098	0.9345
DDX26B	-0.0070	0.7128	0.9352
PRICKLE3	-0.0068	0.7174	0.9367
NLGN4X	-0.0070	0.7184	0.9369
FLNA	-0.0070	0.7233	0.9390
MAMLD1	-0.0067	0.7260	0.9395
TRMT2B	0.0070	0.7297	0.9408
ARMCX1	-0.0065	0.7303	0.9410
ZRSR2	0.0066	0.7321	0.9415
EFNB1	0.0067	0.7339	0.9419
SAT1	-0.0065	0.7348	0.9424
CFP	0.0065	0.7385	0.9434
UBQLN2	-0.0064	0.7465	0.9458
TBC1D25	-0.0063	0.7487	0.9469
RPS4X	0.0059	0.7560	0.9482
HAUS7	-0.0056	0.7632	0.9497
WAS	-0.0056	0.7716	0.9530
MTM1	-0.0052	0.7730	0.9534
LDOC1	0.0054	0.7830	0.9564
SPIN2A	-0.0054	0.7822	0.9564
DMD	-0.0051	0.7895	0.9584
SLITRK2	-0.0050	0.7889	0.9584
HTATSF1	0.0049	0.7960	0.9592
CTPS2	-0.0048	0.7953	0.9592
TCEAL1	0.0048	0.8024	0.9600
REPS2	-0.0048	0.8052	0.9605
TCEAL8	0.0047	0.8052	0.9605
GPR34	-0.0045	0.8080	0.9613
RAB39B	0.0046	0.8090	0.9618
HEPH	0.0045	0.8122	0.9624
FRMPD4	-0.0044	0.8236	0.9652
STS	-0.0044	0.8240	0.9652
DMRTC1	0.0044	0.8250	0.9653
IL3RA	0.0042	0.8258	0.9653
ZC4H2	0.0044	0.8252	0.9653

© 2021 Davis EJ et al. *JAMA Neurology.*

SYAP1	-0.0042	0.8269	0.9655
BCAP31	-0.0042	0.8274	0.9657
WDR44	-0.0040	0.8319	0.9664
TMEM35	0.0039	0.8348	0.9672
DCX	-0.0039	0.8356	0.9672
NUDT11	0.0039	0.8370	0.9675
RIBC1	0.0038	0.8394	0.9683
SHROOM4	0.0039	0.8401	0.9684
TMEM164	-0.0038	0.8404	0.9685
CLCN4	-0.0037	0.8477	0.9696
LAS1L	-0.0036	0.8489	0.9698
RGAG4	-0.0037	0.8486	0.9698
TMEM185A	-0.0036	0.8520	0.9705
TMEM187	0.0033	0.8592	0.9721
PRDX4	0.0033	0.8589	0.9721
TCEAL4	-0.0033	0.8614	0.9726
ARHGAP4	0.0033	0.8625	0.9728
CXorf57	-0.0032	0.8677	0.9740
GTPBP6	-0.0031	0.8673	0.9740
CETN2	-0.0032	0.8681	0.9740
GABRA3	0.0032	0.8680	0.9740
DKC1	0.0031	0.8722	0.9750
FAAH2	-0.0030	0.8729	0.9750
APEX2	-0.0030	0.8752	0.9756
ZNF185	-0.0031	0.8771	0.9759
MAGED4	-0.0030	0.8804	0.9767
TCEAL3	-0.0028	0.8816	0.9771
FAM123B	0.0026	0.8883	0.9786
SASH3	0.0026	0.8889	0.9786
CCDC22	0.0026	0.8937	0.9796
RPL39	0.0025	0.8964	0.9800
NDUFA1	-0.0024	0.9008	0.9810
PAK3	0.0023	0.9015	0.9812
KDM5C	-0.0023	0.9023	0.9813
ZNF41	0.0023	0.9052	0.9816
KIAA2022	-0.0022	0.9091	0.9820
PIN4	0.0020	0.9158	0.9832
CDK16	-0.0020	0.9170	0.9834
IDH3G	-0.0019	0.9178	0.9838
HCCS	0.0019	0.9196	0.9845
L1CAM	-0.0019	0.9196	0.9845
TCEAL2	0.0019	0.9201	0.9846

© 2021 Davis EJ et al. *JAMA Neurology.*

MTMR1	-0.0017	0.9278	0.9859
CD99L2	0.0017	0.9318	0.9861
OFD1	-0.0016	0.9327	0.9861
PNMA6A	0.0017	0.9301	0.9861
ZNF711	0.0016	0.9341	0.9864
ARMCX3	-0.0015	0.9372	0.9872
F8A1	-0.0014	0.9452	0.9892
SNX12	0.0013	0.9456	0.9892
CA5B	0.0013	0.9456	0.9892
HSD17B10	-0.0013	0.9463	0.9892
PJA1	-0.0013	0.9467	0.9892
BGN	-0.0012	0.9483	0.9894
WASH6P	-0.0012	0.9492	0.9894
CHIC1	0.0012	0.9519	0.9899
ZNF275	-0.0011	0.9521	0.9899
TMEM47	0.0011	0.9536	0.9905
IGBP1	0.0010	0.9595	0.9923
SMARCA1	0.0008	0.9657	0.9930
TIMP1	-0.0008	0.9660	0.9931
RNF113A	0.0008	0.9670	0.9932
MAGEC3	0.0008	0.9686	0.9936
CHST7	0.0005	0.9780	0.9956
NUDT10	0.0005	0.9797	0.9960
GSPT2	0.0005	0.9805	0.9960
FMR1	0.0004	0.9834	0.9963
ODZ1	-0.0002	0.9908	0.9977
FTSJ1	-0.0001	0.9972	0.9995
HUWE1	-0.0001	0.9976	0.9995

© 2021 Davis EJ et al. *JAMA Neurology.*

**eTable 6**. **Association of X chromosome gene expression with neurofibrillary tangles in men, adjusted by age and education.** FDR-adjusted p-values for genome-wide correction shown.			
**Gene**	**Beta**	**Pvalue unadjusted**	**Pvalue adjusted**
EMD	-0.0982	0.0000	**0.0266**
UBL4A	0.0975	0.0000	**0.0317**
PHF16	0.0901	0.0000	**0.0320**
PNMA5	-0.0841	0.0003	0.0636
SSR4	-0.0732	0.0006	0.0821
SMC1A	0.0879	0.0006	0.0857
PRAF2	-0.0772	0.0008	0.0949
LAS1L	-0.0761	0.0008	0.0975
TCEAL5	-0.0803	0.0008	0.0981
ZBED1	0.0772	0.0009	0.1022
FOXO4	0.0730	0.0012	0.1119
RPL10	-0.0749	0.0013	0.1156
FAM156B	-0.0736	0.0015	0.1247
ARMCX4	-0.0709	0.0016	0.1283
ZMAT1	-0.0698	0.0020	0.1410
FAM127A	-0.0673	0.0021	0.1419
GYG2	0.0732	0.0028	0.1603
POLA1	0.0728	0.0031	0.1671
HCFC1	0.0698	0.0034	0.1735
FAM123B	0.0670	0.0039	0.1793
GEMIN8	-0.0643	0.0060	0.2095
HUWE1	0.0649	0.0060	0.2099
PHF6	0.0637	0.0069	0.2175
GK	-0.0658	0.0071	0.2206
IRAK1	0.0616	0.0071	0.2206
PDZD11	-0.0607	0.0072	0.2215
BEX4	-0.0602	0.0083	0.2334
SLC35A2	-0.0590	0.0085	0.2341
HAUS7	-0.0613	0.0093	0.2383
F8A3	0.0563	0.0102	0.2476
TSPAN7	-0.0555	0.0112	0.2561
NAA10	-0.0576	0.0114	0.2567
ARHGAP4	-0.0576	0.0119	0.2608
EBP	-0.0591	0.0124	0.2623
RBMX2	-0.0581	0.0127	0.2645
MTCP1NB	-0.0585	0.0135	0.2681
OFD1	-0.0554	0.0137	0.2703
CLCN4	0.0556	0.0138	0.2709
RAB9A	0.0563	0.0140	0.2722

© 2021 Davis EJ et al. *JAMA Neurology.*

F8A2	0.0539	0.0144	0.2743
SMARCA1	0.0588	0.0145	0.2746
TRMT2B	0.0522	0.0148	0.2776
RIBC1	-0.0568	0.0154	0.2798
RAI2	0.0549	0.0157	0.2818
BCORL1	0.0583	0.0158	0.2821
FAM155B	0.0532	0.0164	0.2841
ZNF185	-0.0514	0.0169	0.2876
GPR143	-0.0547	0.0169	0.2876
CCDC160	0.0572	0.0174	0.2897
BEX2	-0.0542	0.0183	0.2915
APLN	0.0580	0.0186	0.2915
FUNDC1	-0.0552	0.0188	0.2924
FAM127B	-0.0513	0.0190	0.2940
MID1IP1	0.0519	0.0192	0.2957
MED14	-0.0522	0.0209	0.3055
GLA	-0.0524	0.0225	0.3144
CHIC1	0.0510	0.0245	0.3253
TAF9B	-0.0525	0.0267	0.3335
DDX26B	-0.0508	0.0277	0.3384
TCEAL2	-0.0501	0.0292	0.3445
FRMPD3	0.0485	0.0308	0.3505
STAG2	0.0471	0.0309	0.3511
CFP	-0.0477	0.0320	0.3542
NKAP	-0.0534	0.0348	0.3636
KLHL13	0.0492	0.0363	0.3693
PGRMC1	-0.0453	0.0391	0.3762
MSL3	0.0470	0.0392	0.3765
F8A1	-0.0427	0.0402	0.3785
CSTF2	-0.0461	0.0418	0.3827
TIMM17B	-0.0511	0.0417	0.3827
PDZD4	0.0458	0.0423	0.3844
TCEAL4	-0.0453	0.0444	0.3915
YIPF6	0.0471	0.0450	0.3928
BHLHB9	0.0457	0.0451	0.3930
ARSD	-0.0461	0.0453	0.3939
WAS	-0.0457	0.0458	0.3950
PNMA3	-0.0460	0.0487	0.4023
MECP2	0.0436	0.0501	0.4066
CCDC120	0.0472	0.0508	0.4088
G6PD	-0.0439	0.0549	0.4205
ASMTL	-0.0439	0.0572	0.4210

© 2021 Davis EJ et al. *JAMA Neurology.*

FAM120C	0.0473	0.0587	0.4238
TSC22D3	0.0437	0.0598	0.4276
ZBTB33	0.0439	0.0598	0.4278
NDUFA1	-0.0427	0.0600	0.4281
RPL36A	-0.0423	0.0641	0.4390
RRAGB	-0.0428	0.0646	0.4402
CETN2	-0.0425	0.0651	0.4413
KLHL4	0.0418	0.0657	0.4422
TFE3	0.0448	0.0658	0.4426
PTCHD1	0.0417	0.0660	0.4432
WWC3	0.0391	0.0664	0.4437
PHKA2	-0.0405	0.0666	0.4440
MCF2	0.0416	0.0727	0.4551
ZNF673	-0.0398	0.0734	0.4568
USP11	-0.0414	0.0752	0.4605
RPS6KA3	0.0400	0.0753	0.4608
DMRTC1	-0.0383	0.0762	0.4625
TMEM185A	0.0394	0.0762	0.4625
ELK1	0.0411	0.0774	0.4644
RHOXF1	-0.0356	0.0775	0.4644
PIN4	-0.0394	0.0775	0.4644
IKBKG	-0.0402	0.0804	0.4713
BCOR	0.0412	0.0835	0.4782
RPGR	-0.0396	0.0847	0.4799
ZCCHC12	-0.0392	0.0868	0.4848
HPRT1	-0.0402	0.0892	0.4902
TXLNG	-0.0367	0.0888	0.4902
SLC25A53	0.0382	0.0897	0.4910
PIR	-0.0359	0.0897	0.4910
TCEAL7	-0.0379	0.0902	0.4924
NHS	0.0392	0.0915	0.4950
MOSPD1	0.0403	0.0921	0.4952
ZNF674	0.0388	0.0952	0.5017
HMGB3	-0.0381	0.0956	0.5028
SPIN2B	-0.0402	0.0960	0.5036
LDOC1	-0.0361	0.0960	0.5036
PPP1R3F	0.0398	0.0961	0.5037
BEX1	-0.0385	0.0985	0.5074
CA5B	-0.0367	0.1005	0.5101
ACSL4	-0.0365	0.1050	0.5179
LONRF3	0.0371	0.1059	0.5185
DDX3X	0.0418	0.1064	0.5189

© 2021 Davis EJ et al. *JAMA Neurology.*

TCEANC	0.0348	0.1074	0.5191
TMLHE	-0.0379	0.1075	0.5192
C1GALT1C1	0.0386	0.1169	0.5335
AIFM1	-0.0358	0.1178	0.5353
PNCK	-0.0358	0.1219	0.5404
FLNA	-0.0340	0.1239	0.5428
ZNF630	0.0346	0.1265	0.5476
ARMCX5	-0.0347	0.1276	0.5495
ARAF	-0.0356	0.1288	0.5512
CXorf23	-0.0356	0.1299	0.5536
IDH3G	-0.0351	0.1328	0.5582
NLGN4X	0.0341	0.1347	0.5605
CXorf56	-0.0336	0.1393	0.5656
HTATSF1	0.0339	0.1404	0.5665
PHF8	0.0343	0.1428	0.5696
TBC1D25	0.0323	0.1437	0.5712
APOO	-0.0352	0.1442	0.5720
ZFX	0.0340	0.1443	0.5721
HDHD1	0.0313	0.1498	0.5781
UXT	0.0323	0.1531	0.5822
TAF1	-0.0356	0.1543	0.5824
TSPYL2	-0.0314	0.1616	0.5911
BEX5	-0.0340	0.1621	0.5918
RBM10	-0.0311	0.1624	0.5923
ATRX	0.0342	0.1672	0.5980
DNASE1L1	-0.0291	0.1731	0.6037
GPKOW	-0.0349	0.1732	0.6037
TCEAL6	-0.0311	0.1736	0.6045
ARMCX6	-0.0317	0.1745	0.6060
REPS2	0.0311	0.1746	0.6061
KDM5C	0.0328	0.1780	0.6105
PDHA1	-0.0289	0.1801	0.6132
HCCS	0.0309	0.1810	0.6136
NDUFB11	0.0307	0.1820	0.6144
PORCN	-0.0297	0.1886	0.6194
RBMX	-0.0298	0.1889	0.6196
MAOA	-0.0303	0.1945	0.6257
ENOX2	0.0291	0.1945	0.6257
ZNF275	0.0301	0.1949	0.6258
FAM70A	0.0294	0.1973	0.6282
COL4A5	-0.0295	0.2072	0.6376
ARMCX3	0.0291	0.2098	0.6403

© 2021 Davis EJ et al. *JAMA Neurology.*

FRMPD4	0.0283	0.2101	0.6406
SCML1	-0.0288	0.2121	0.6433
APEX2	0.0290	0.2171	0.6483
GPRASP1	-0.0291	0.2178	0.6487
CASK	0.0282	0.2211	0.6510
ARHGEF6	0.0305	0.2255	0.6552
NAP1L3	-0.0278	0.2274	0.6562
SMS	-0.0278	0.2293	0.6580
APOOL	0.0281	0.2315	0.6601
WBP5	0.0273	0.2340	0.6628
GRIA3	-0.0261	0.2365	0.6652
HDAC6	-0.0281	0.2402	0.6681
IL3RA	0.0265	0.2417	0.6693
ZNF41	0.0261	0.2433	0.6708
CHRDL1	-0.0277	0.2443	0.6718
SPIN2A	0.0262	0.2463	0.6740
MAOB	-0.0296	0.2470	0.6749
HNRNPH2	-0.0269	0.2497	0.6776
GABRA3	0.0258	0.2590	0.6849
WDR44	0.0265	0.2597	0.6854
ARSE	0.0265	0.2625	0.6876
PIM2	-0.0252	0.2625	0.6876
ZC4H2	-0.0247	0.2640	0.6882
ALG13	-0.0255	0.2681	0.6916
TBC1D8B	-0.0246	0.2685	0.6918
ATP6AP2	-0.0259	0.2707	0.6936
FMR1	0.0263	0.2710	0.6938
GABRE	-0.0257	0.2723	0.6954
TMSB15B	-0.0248	0.2723	0.6954
CITED1	-0.0271	0.2742	0.6970
CD99L2	0.0237	0.2812	0.7028
RBM3	-0.0250	0.2811	0.7028
VMA21	-0.0256	0.2828	0.7041
IL13RA1	-0.0253	0.2834	0.7046
DMD	-0.0242	0.2836	0.7046
TSR2	-0.0249	0.2841	0.7050
THOC2	-0.0258	0.2860	0.7060
RP1-177G6.2	-0.0256	0.2868	0.7066
NGFRAP1	-0.0248	0.2905	0.7094
ZNF711	0.0242	0.2922	0.7108
DHRSX	-0.0247	0.2921	0.7108
BTK	-0.0252	0.2920	0.7108

© 2021 Davis EJ et al. *JAMA Neurology.*

SCML2	0.0264	0.2937	0.7116
MID2	0.0261	0.2937	0.7116
ZMYM3	0.0252	0.2952	0.7129
COX7B	-0.0229	0.2986	0.7164
SHROOM2	0.0228	0.2989	0.7167
RNF128	-0.0246	0.3001	0.7172
FAM58A	0.0250	0.3007	0.7172
ACRC	0.0251	0.3059	0.7211
MORF4L2	-0.0235	0.3065	0.7214
MBTPS2	-0.0234	0.3073	0.7220
FAM104B	-0.0238	0.3078	0.7221
TIMP1	-0.0242	0.3083	0.7224
FUNDC2	0.0238	0.3090	0.7227
KIAA2022	0.0235	0.3098	0.7231
ERAS	0.0235	0.3097	0.7231
FAM122B	0.0230	0.3171	0.7291
MAGT1	-0.0225	0.3195	0.7313
F8	-0.0219	0.3206	0.7313
CXorf1	0.0223	0.3224	0.7328
PLCXD1	0.0246	0.3256	0.7348
TMEM187	0.0230	0.3284	0.7359
AP1S2	0.0230	0.3294	0.7366
PSMD10	-0.0238	0.3345	0.7401
PRRG3	-0.0207	0.3397	0.7435
KCND1	0.0205	0.3407	0.7445
IQSEC2	0.0222	0.3412	0.7449
LAMP2	0.0219	0.3454	0.7477
LRCH2	0.0208	0.3467	0.7486
SNX12	0.0209	0.3557	0.7548
MSN	-0.0231	0.3573	0.7555
CSAG1	0.0204	0.3599	0.7576
BCAP31	0.0210	0.3606	0.7577
VAMP7	0.0221	0.3627	0.7593
WASH6P	0.0210	0.3640	0.7602
RNF113A	0.0198	0.3651	0.7608
PDK3	-0.0206	0.3668	0.7610
CXorf57	-0.0211	0.3720	0.7641
MAGED4B	0.0212	0.3730	0.7647
TMSB4X	-0.0223	0.3743	0.7650
DACH2	-0.0209	0.3782	0.7680
KAL1	-0.0205	0.3821	0.7703
RPL39	-0.0207	0.3822	0.7704

© 2021 Davis EJ et al. *JAMA Neurology.*

HMGN5	0.0205	0.3839	0.7717
UPRT	0.0197	0.3896	0.7757
HSFX1	-0.0205	0.3912	0.7769
ATP11C	0.0208	0.3966	0.7805
RGN	-0.0192	0.4008	0.7825
DKC1	0.0189	0.4018	0.7832
DMRTC1B	-0.0197	0.4026	0.7836
UTP14A	-0.0181	0.4031	0.7838
FAM127C	-0.0174	0.4181	0.7927
SYN1	-0.0179	0.4200	0.7932
PNPLA4	-0.0192	0.4214	0.7940
SLC25A14	-0.0185	0.4254	0.7959
MORC4	0.0183	0.4266	0.7970
TAB3	0.0176	0.4320	0.8000
TRAPPC2	0.0184	0.4340	0.8011
ZDHHC9	-0.0189	0.4383	0.8038
GDI1	-0.0189	0.4425	0.8069
PIGA	-0.0184	0.4426	0.8070
WDR45	0.0182	0.4499	0.8111
DLG3	0.0171	0.4551	0.8130
RBM41	0.0171	0.4573	0.8138
OCRL	0.0164	0.4599	0.8147
RAB33A	0.0162	0.4605	0.8150
DIAPH2	-0.0159	0.4620	0.8165
GPR173	0.0174	0.4634	0.8174
TSPAN6	-0.0167	0.4639	0.8176
PLS3	-0.0173	0.4646	0.8179
CHM	0.0163	0.4680	0.8200
FAM133A	-0.0183	0.4722	0.8225
DYNLT3	-0.0173	0.4728	0.8227
PRRG1	0.0161	0.4826	0.8291
HDAC8	-0.0156	0.4844	0.8304
SLITRK4	0.0155	0.4857	0.8314
IL1RAPL1	-0.0175	0.4875	0.8323
ZNF75D	-0.0155	0.4870	0.8323
AL158821.1	-0.0155	0.4901	0.8326
CUL4B	0.0165	0.4911	0.8330
DRP2	-0.0158	0.4944	0.8343
GNL3L	0.0157	0.4950	0.8344
MAGEH1	-0.0155	0.4969	0.8357
SASH3	-0.0169	0.5000	0.8380
INE1	-0.0160	0.5069	0.8405

© 2021 Davis EJ et al. *JAMA Neurology.*

CYBB	-0.0167	0.5080	0.8413
PPEF1	-0.0158	0.5092	0.8415
MCTS1	-0.0157	0.5110	0.8428
RPS4X	-0.0154	0.5137	0.8438
SUV39H1	0.0149	0.5151	0.8440
NUDT10	0.0150	0.5163	0.8446
AFF2	0.0142	0.5177	0.8446
VBP1	-0.0141	0.5181	0.8447
ARX	0.0146	0.5183	0.8447
SLC25A43	-0.0139	0.5205	0.8453
SLC38A5	-0.0157	0.5202	0.8453
RGAG4	0.0146	0.5219	0.8462
PABPC1L2A	0.0142	0.5269	0.8486
PLP2	-0.0146	0.5296	0.8501
MAP7D3	0.0145	0.5318	0.8510
MUM1L1	0.0140	0.5336	0.8514
IGSF1	-0.0140	0.5345	0.8518
CLIC2	0.0144	0.5447	0.8578
NDP	0.0144	0.5462	0.8585
ABCD1	0.0131	0.5493	0.8604
NSDHL	-0.0137	0.5515	0.8611
MAGEC3	-0.0122	0.5548	0.8619
CXorf38	-0.0149	0.5559	0.8624
FAM122C	-0.0147	0.5583	0.8639
PRPS2	-0.0132	0.5597	0.8645
FGD1	0.0132	0.5619	0.8654
SLC9A7	0.0127	0.5622	0.8656
MAGIX	-0.0137	0.5676	0.8678
STARD8	0.0135	0.5700	0.8689
USP9X	0.0144	0.5729	0.8701
TCEAL3	-0.0128	0.5763	0.8715
IDS	0.0128	0.5763	0.8715
ZC3H12B	0.0127	0.5842	0.8756
NONO	0.0138	0.5868	0.8768
CSF2RA	-0.0119	0.5880	0.8774
L1CAM	0.0127	0.5899	0.8782
ATG4A	0.0123	0.5936	0.8795
ZNF449	-0.0123	0.5955	0.8803
NXT2	0.0122	0.5975	0.8812
UPF3B	-0.0134	0.5975	0.8812
SRPX	-0.0119	0.5990	0.8812
CNKSR2	0.0119	0.5996	0.8815

© 2021 Davis EJ et al. *JAMA Neurology.*

MID1	-0.0126	0.6044	0.8829
PLXNA3	-0.0113	0.6094	0.8857
SH3KBP1	0.0108	0.6111	0.8866
PCSK1N	-0.0124	0.6126	0.8873
CDK16	0.0118	0.6125	0.8873
ITM2A	-0.0118	0.6126	0.8873
PNMA6C	0.0103	0.6150	0.8882
KCNE1L	0.0103	0.6164	0.8890
SAT1	-0.0114	0.6197	0.8898
RP11-706O15.1	0.0125	0.6227	0.8906
TBL1X	0.0119	0.6230	0.8908
XIAP	0.0113	0.6340	0.8952
AKAP17A	-0.0107	0.6355	0.8962
XK	-0.0108	0.6361	0.8964
PLXNB3	-0.0107	0.6435	0.8995
HS6ST2	0.0104	0.6439	0.8998
GPC4	0.0116	0.6456	0.9002
EIF2S3	-0.0104	0.6468	0.9006
SH3BGRL	0.0115	0.6480	0.9009
MAMLD1	-0.0105	0.6493	0.9016
ARMCX1	-0.0107	0.6499	0.9019
AMOT	0.0106	0.6547	0.9038
GPRASP2	-0.0100	0.6549	0.9039
CD99	-0.0105	0.6550	0.9040
RAB39B	0.0102	0.6559	0.9044
SYTL4	0.0106	0.6582	0.9050
GDPD2	-0.0105	0.6613	0.9066
MOSPD2	0.0098	0.6625	0.9073
SMPX	-0.0101	0.6640	0.9077
SRPK3	0.0100	0.6664	0.9085
BRWD3	0.0099	0.6666	0.9086
IL2RG	0.0111	0.6687	0.9094
DCX	0.0103	0.6727	0.9111
IGBP1	0.0092	0.6763	0.9127
PLP1	-0.0098	0.6761	0.9127
EIF1AX	0.0098	0.6760	0.9127
SLC25A6	0.0094	0.6767	0.9129
EFNB1	0.0090	0.6819	0.9147
UBQLN2	-0.0085	0.6945	0.9191
PCYT1B	0.0093	0.6948	0.9191
OPHN1	-0.0096	0.6939	0.9191
PJA1	-0.0092	0.6946	0.9191

© 2021 Davis EJ et al. *JAMA Neurology.*

MPP1	0.0088	0.6984	0.9198
CXorf40B	-0.0085	0.7054	0.9217
SPIN3	-0.0084	0.7188	0.9264
TMEM35	0.0085	0.7192	0.9265
PCDH11X	0.0080	0.7207	0.9271
RLIM	0.0083	0.7222	0.9278
ZDHHC15	0.0077	0.7252	0.9285
ARHGEF9	-0.0079	0.7269	0.9292
CXorf69	-0.0084	0.7303	0.9299
ARSF	0.0083	0.7313	0.9299
PGK1	0.0084	0.7331	0.9304
ACOT9	-0.0074	0.7449	0.9348
GPR34	-0.0077	0.7460	0.9351
PRKX	0.0070	0.7470	0.9353
OTUD5	-0.0069	0.7503	0.9353
TAZ	0.0073	0.7487	0.9353
MAGEE1	-0.0073	0.7544	0.9354
ATP6AP1	-0.0068	0.7606	0.9365
CYSLTR1	-0.0071	0.7610	0.9365
FAM156A	-0.0069	0.7619	0.9369
BRCC3	-0.0068	0.7649	0.9383
KDM6A	0.0076	0.7701	0.9390
ZNF182	-0.0064	0.7722	0.9390
NAP1L2	-0.0067	0.7761	0.9394
FHL1	-0.0070	0.7765	0.9394
CDKL5	-0.0065	0.7773	0.9398
SYP	-0.0066	0.7805	0.9409
UBA1	-0.0064	0.7892	0.9440
UBE2A	-0.0061	0.7900	0.9443
DUSP9	-0.0064	0.7907	0.9446
MTCP1	-0.0064	0.7916	0.9448
FTSJ1	-0.0062	0.7984	0.9477
HEPH	-0.0058	0.8057	0.9498
MTM1	-0.0061	0.8087	0.9509
ZXDB	-0.0055	0.8099	0.9510
PCDH19	-0.0060	0.8099	0.9510
PHKA1	0.0055	0.8106	0.9510
MAGED4	0.0052	0.8132	0.9515
MAGED2	0.0055	0.8143	0.9520
SLITRK2	-0.0055	0.8144	0.9520
VSIG4	-0.0058	0.8165	0.9525
CCDC22	0.0050	0.8211	0.9538

© 2021 Davis EJ et al. *JAMA Neurology.*

CHST7	0.0049	0.8229	0.9543
NLGN3	0.0054	0.8229	0.9543
OGT	0.0051	0.8324	0.9573
RENBP	0.0049	0.8322	0.9573
SLC9A6	-0.0046	0.8370	0.9589
FAM199X	0.0048	0.8386	0.9593
MAGEE2	-0.0045	0.8401	0.9596
CXorf26	-0.0045	0.8419	0.9601
GSPT2	0.0043	0.8423	0.9603
MAP7D2	0.0047	0.8444	0.9608
ARMCX2	-0.0044	0.8479	0.9618
KLF8	-0.0047	0.8482	0.9618
CXorf40A	0.0041	0.8505	0.9618
PQBP1	0.0042	0.8507	0.9619
ZNF81	0.0042	0.8535	0.9626
SLC6A8	0.0043	0.8544	0.9627
RP11-274K13.2	-0.0040	0.8580	0.9641
NKRF	0.0041	0.8592	0.9644
CXorf36	0.0041	0.8608	0.9650
AMMECR1	0.0041	0.8611	0.9651
FIGF	-0.0039	0.8627	0.9656
FAM3A	-0.0040	0.8664	0.9666
SYAP1	0.0038	0.8670	0.9667
ODZ1	-0.0040	0.8698	0.9673
FGF13	-0.0037	0.8724	0.9679
HSD17B10	0.0038	0.8734	0.9682
KLHL15	0.0034	0.8822	0.9710
BGN	0.0033	0.8869	0.9721
SLC16A2	0.0034	0.8887	0.9723
LAGE3	-0.0032	0.8935	0.9733
TCEAL8	0.0031	0.8943	0.9734
EFHC2	0.0031	0.8951	0.9739
FATE1	-0.0028	0.8961	0.9744
RAB9B	-0.0028	0.8997	0.9757
TCEAL1	0.0028	0.8993	0.9757
SEPT	-0.0028	0.9018	0.9761
MAGED1	-0.0029	0.9016	0.9761
USP51	-0.0028	0.9093	0.9782
GTPBP6	-0.0028	0.9097	0.9783
CXorf24	-0.0023	0.9136	0.9796
TMEM47	-0.0024	0.9150	0.9802
TRO	0.0025	0.9182	0.9812

© 2021 Davis EJ et al. *JAMA Neurology.*

RBBP7	0.0020	0.9256	0.9835
PPP2R3B	-0.0022	0.9258	0.9836
STS	0.0020	0.9281	0.9839
MTMR1	-0.0022	0.9281	0.9839
PABPC1L2B	0.0019	0.9344	0.9850
TMEM164	0.0017	0.9413	0.9870
TIMM8A	-0.0015	0.9445	0.9881
GPM6B	-0.0016	0.9442	0.9881
GRIPAP1	0.0015	0.9469	0.9882
MMGT1	0.0015	0.9491	0.9885
CTPS2	-0.0015	0.9504	0.9888
NUDT11	-0.0015	0.9509	0.9888
ZRSR2	-0.0013	0.9539	0.9895
GJB1	0.0013	0.9542	0.9896
FAAH2	-0.0012	0.9588	0.9908
PNMA6A	0.0009	0.9681	0.9928
PRDX4	0.0010	0.9688	0.9929
MED12	-0.0008	0.9701	0.9930
ABCB7	0.0010	0.9699	0.9930
ATP2B3	-0.0008	0.9726	0.9936
SHROOM4	0.0008	0.9721	0.9936
PAK3	0.0008	0.9734	0.9937
PRICKLE3	-0.0008	0.9738	0.9938
FAM50A	-0.0007	0.9765	0.9947
RAP2C	-0.0006	0.9776	0.9949
WDR13	-0.0005	0.9823	0.9962
PRPS1	-0.0004	0.9877	0.9974
SLC10A3	-0.0003	0.9893	0.9982
SLC25A5	-0.0002	0.9945	0.9990

© 2021 Davis EJ et al. *JAMA Neurology.*
